# Targeted in vivo knock-in of human alpha-1-antitrypsin cDNA using adenoviral delivery of CRISPR/Cas9

**DOI:** 10.1038/s41434-018-0003-1

**Published:** 2018-03-27

**Authors:** Calvin J. Stephens, Elena Kashentseva, William Everett, Lyudmila Kaliberova, David T. Curiel

**Affiliations:** 1 Department of Radiation Oncology, Cancer Biology Division, School of Medicine, Washington University in Saint Louis, 660 South Euclid Avenue, Campus Box 8224, St. Louis, MO 63110 USA; 2 Molecular Genetics and Genomics Program, Division of Biology and Biomedical Sciences, School of Medicine, Washington University in Saint Louis, 660 South Euclid Avenue, Campus Box 8226, St. Louis, MO 63110 USA; 3 Department of Radiation Oncology, Biologic Therapeutics Center, School of Medicine, Washington University in Saint Louis, 660 South Euclid Avenue, Campus Box 8224, St. Louis, MO 63110 USA

## Abstract

Serum deficiency diseases such as alpha-1-antitrypsin deficiency are characterized by reduced function of serum proteins, caused by deleterious genetic mutations. These diseases are promising targets for genetic interventions. Gene therapies using viral vectors have been used to introduce correct copies of the disease-causing gene in preclinical and clinical studies. However, these studies highlighted that disease-alleviating gene expression is lost over time. Integration into a specific chromosomal site could provide lasting therapeutic expression to overcome this major limitation. Additionally, targeted integration could avoid detrimental mutagenesis associated with integrative vectors, such as tumorigenesis or functional gene perturbation. To test if adenoviral vectors can facilitate long-term gene expression through targeted integration, we somatically incorporated the human alpha-1-antitrypsin gene into the *ROSA26* “safe harbor” locus in murine livers, using CRISPR/Cas9. We found adenoviral-mediated delivery of CRISPR/Cas9 achieved gene editing outcomes persisting over 200 days. Furthermore, gene knock-in maintained greater levels of the serum protein than provided by episomal expression. Importantly, our “knock-in” approach is generalizable to other serum proteins and supports in vivo cDNA replacement therapy to achieve stable gene expression.

## Introduction

Gene therapy can potentially be a curative treatment for inherited metabolic disorders, including serum deficiency diseases. Such interventions for serum deficiencies employ strategies to introduce a corrected copy of the deficient gene. Initial gene therapies have used retroviral and lentiviral vectors to integrate the correct allele copy into the chromosomes of animal models, and human subjects, to provide corrective factor expression [1]. Utilization of these vectors in clinical settings, however, has been limited by scaling issues, cost, and difficulty of production [2, 3]. Additionally, safety concerns from genotoxicity and oncogene activation, caused by the semi-random integration of these vectors, remain relevant [4, 5]. These issues have led to the exploration of therapies based upon non-integrative vectors, such as adeno-associated virus (AAV). Nonetheless, whereas the majority of therapeutic AAV-based gene expression is derived from episomal vector genomes, a fraction of AAV genomes will in fact randomly integrate into the host chromosome [6, 7]. Notably, AAV-based treatment of selected plasma deficiency disorders successfully ameliorated disease phenotype in human clinical trials [8]. In this context, transient therapeutic gene expression, due to the loss of vector genomes through host immune response and cell division, remains a limiting issue. These interventions have thus illustrated the obstacles which must be overcome before fully curative gene therapies are achieved [9, 10].

In this regard, the integration of therapeutic transgenes in a specific location within a chromosome, through targeted gene editing, represents a promising strategy to traverse the limitation of transient gene expression. In addition, a targeted integration therapy could potentially allow extended gene expression without the risk of insertional mutagenesis associated with available integrating viral vectors. Initial viral vector-based gene editing approaches have used zinc finger nucleases to induce targeted homologous recombination, showing that correction of a hemophilia disease phenotype is possible using designer nucleases [11]. Subsequently, other studies have employed gene editing, with and without designer nucleases, to successfully treat hemophilia [12–15]. Several other proof-of-principle studies have also shown the in vivo therapeutic benefits that may accrue from viral vector-mediated gene editing with CRISPR/Cas9, supporting the feasibility of gene editing for an extended range of disease targets [16, 17].

Nonetheless, practical employment of such CRISPR/Cas9 strategies are ultimately subservient to the in vivo efficiencies of available vector systems. In this regard, it remains unclear whether the CRISPR/Cas9 system can be efficiently delivered in vivo to enough cells, or target organs, to treat the range of clinical syndromes caused by serum protein deficiencies. Of note, these disorders will require high-efficiency gene transfer, and editing activity, to achieve therapeutic outcomes. On this basis, we explored adenoviral vectors in combination with CRISPR/Cas9 gene editing. This choice was based on the recognized utility of adenovirus in achieving in vivo transduction at high levels of efficiency [18, 19].

In this proof-of-principle study, we show efficient adenoviral-mediated delivery of CRISPR/Cas9 achieved successful targeted “knock-in” of *hAAT* and *EGFP* genes at the *ROSA26* safe harbor locus in murine livers. Most significantly, *hAAT* knock-in enabled long-term augmentation of serum hAAT levels in mice. Our study thus establishes adenovirus in vivo delivery of CRISPR/Cas9 can achieve long-term expression of serum protein relevant to a deficiency disorder. This study thus highlights the utilities of adenoviral vector-mediated gene delivery in feasibilizing in vivo gene knock-in. Such an approach potentially provides a generalizable method to realize long-term gene expression from this useful class of vectors.

## Materials and methods

### Cell culture

Human embryonic kidney 293 (HEK293) cells (Microbix Biosystems Inc., Mississauga, ON, Canada) were cultured in sterile conditions with Dulbecco’s modified essential media-F12 Mix (DMEM-F12) (Lonza-BioWhittaker, Basel, Switzerland) supplemented with 10% fetal bovine serum (FBS) (Sigma-Aldrich, St. Louis, MO, USA), or minimal essential media (Corning Inc., Corning, NY, USA) with 2% FBS during viral rescue. Human A549 cells (ATTC, Manassas, VA, USA) and murine liver cell line BNL-1NG (ATCC) were cultured in 10% FBS DMEM-F12.

### Mouse experiments

Male C57Bl/6J (Jackson Laboratory, Bar Harbor, ME, USA) mice were housed in an approved animal housing facility. All experiments were performed in strict accordance with the Guide for the Care and Use of Laboratory Animals of the National Institutes of Health and with a pre-approved Institutional Animal Care and Use Committee protocol (#201510191). All animals were housed under pathogen-free conditions with access to chow and water ad libitum. Intravenous injections were administered through the tail vein at 4 weeks of age. Blood collections were performed through facial vein puncture, into heparin-coated capillaries. Plasma was aliquoted and stored at −80 °C. Animals were humanely sacrificed by CO_2_ inhalation; major organs were harvested and snap frozen on dry ice.

### Vector construction

**Fig. 1:**
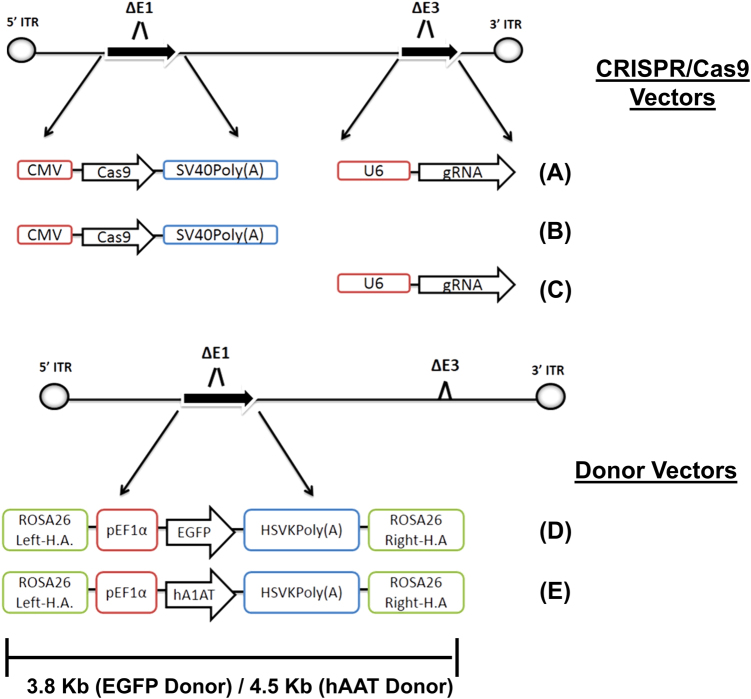
Adenoviral-mediated delivery of CRISPR/Cas9 for targeted gene editing. Genetic designs of a CRISPR/Cas9-based strategy for targeted homologous recombination. Ad5-Cas9-gRNA (**a**) expresses both the *Cas9* gene and a *ROSA26-*targeting gRNA sequence from the E1- and the E3-deleted regions, respectively. Ad5-Cas9 (**b**) expresses the *Cas9* gene from the E1-deleted region. Ad5-gRNA (**c**) contains an E1-deleted region and expresses the *ROSA26* gRNA from the E3-deleted region. Ad5-EF1α-EGFP (**d**) expresses the *EGFP* gene from the mammalian EF1α promoter/intron and contains 0.8 kb length homology arms to the *ROSA26* locus surrounding the expression cassette, in the E1-deleted region. Ad5-EF1α-hAAT (**e**) expresses the *hAAT* gene from the same expression cassette and homology arms as the EGFP donor vector

Viral construction was completed using three different plasmids. Each contained either the gene for *Streptococcus pyrogenes* Cas9, a 20-nucleotide (nt) *ROSA26-*targeting guide RNA (gRNA), or a *ROSA26* homology donor cassette, gifted from the Genome Engineering Core (GEiC) at the Washington University in St. Louis (WUSTL). The *Cas9* gene was cloned from the first plasmid into the *Eco*RV site of pShuttle-CMV (cytomegalovirus) (Agilent Technologies, Santa Clara, CA, USA). pShuttle-CMV-Cas9 was recombined into the E1 region of E1-deleted and E3-deleted Ad plasmid DNA, pAdEasy1 (Agilent Technologies) in *Escherichia coli* strain BJ5183 (Agilent Technologies), resulting in pAd5-Cas9. The gRNA targeting the second intronic region of *ROSA26* (chromosome 6 position—113,076,010; targeting sequence: 5′-GGTCTTCTGAGGGCGGGTAGAAG-3′), expressed from the U6 promoter driven by RNA polymerase III, was cloned into the *Bam*HI and *Sal*I sites of plasmid pShuttlE3 [20], in order to construct pShuttlE3-U6-gRNA. pShuttleE3-U6-gRNA was recombined into pAdEasy1 as above, resulting in pAd5-gRNA. Next, pShuttle-CMV-Cas9 was recombined with pAd5-gRNA to obtain a plasmid expressing both CRISPR components, pAd5-Cas9-gRNA (Fig. 1).

Last, donor vectors were constructed using a plasmid containing homology arms (HAs) comprising left and right sequences (800 bp) surrounding the ROSA26 gRNA target site, modified by a multiple cloning site which abolishes gRNA recognition (Fig. 1). Separately, the genes for human *AAT* (Vigene Biosciences Inc., Rockville, MD,USA) and *EGFP* (Takara Bio Inc., Shiga, Japan) were subcloned into *Bam*HI and *Hin*dIII sites of pBApro-EF1α (Clontech Laboratories, Mountain View, CA, USA) containing the constitutive mammalian EF1α promoter and intron. This cloning resulted in pBApro-EF1α-hAAT and pBApro-EF1α-EGFP, respectively. The expression cassettes containing promoter, transgene, and polyA from pBApro-EF1α-hAAT and pBApro-EF1α-EGFP were separately ligated into the above *ROSA26* donor plasmid at the *Pme*I restriction site in negative orientation to endogenous *ROSA26*.

DNA fragments extracted from the resultant donor plasmids for ROSA26-EF1α-hAAT donor and ROSA26-EF1α-EGFP expression cassettes flanked by the *ROSA26* HAs were cloned into the *Eco*RV site of promoterless pShuttle (Agilent Technologies), resulting in two donor plasmids, pShuttle-EF1a-hAAT and pShuttle-EF1a-EGFP. Donor shuttle plasmids were recombined into the E1-deleted region of pAdEasy1, resulting in pAd5-EF1α-EGFP and pAd5-EF1α-hAAT, respectively (Figure 1).

### Recombinant adenovirus (Type-5) vectors

Adenoviruses were produced using standard procedures [21]. Recombinant Ad plasmids were digested with *Pac*I (New England Biolabs, Ipswich, MA, USA), releasing the viral genome, and transfected into HEK293 cells for rescue. We generated five vectors for this study: Ad5-Cas9, Ad5-gRNA, Ad5-Cas9-gRNA, Ad5-EF1α-EGFP, and Ad5-EF1α-hAAT. Following serial amplification, virus was purified with CsCl (Research Products International, Mount Prospect, IL, USA) gradients, dialyzed against phosphate-buffered saline (PBS) (Sigma-Aldrich) containing 10% glycerol (Pharmco Products Inc., Shelbyville, KY, USA) and validated with partial sequence analysis. Viral preparations were titered as viral particles (VP)/mL using the classical Maizel’s method via measurement of A260 absorbance of purified virus particles, with the assumption that 1.1 × 10^12^ VP/mL has an absorbance of 1.0 at 260 nm [22]. Physical vector titer was chosen to reconcile experimental results with prior vectorological studies. Vector transgene expression was validated by Western blot (hAAT and Cas9) and fluorescent microscopy (EGFP) after infection of human A549 or murine BNL-1NG cells. Quality control tests for replication-competent adenovirus were performed using titrations on A549 monolayers, to ensure no replicative virus contaminated preparations.

### In vitro model of integrative and non-integrative vector dilution

Murine liver cell line BNL-1NG was plated on 96-well plates, grown to 70% confluency, and cell numbers were counted. Then, cells in individual wells were infected as follows: (i) PBS, (ii) 10^4^ VP per cell Ad5-sham expressing an irrelevant transgene, (iii) 4 × 10^3^ VP per cell Ad5-Cas9 + 4 × 10^3^ Ad5-EF1α-EGFP, (iv) 6 × 10^3^ VP per cell Ad5-Cas9 + 6 × 10^3^ Ad5-EF1α-EGFP, (v) 4 × 10^3^ VP per cell Ad5-Cas9-gRNA + 4 × 10^3^ Ad5-EF1α-EGFP, and (vi) 6 × 10^3^ VP per cell Ad5-Cas9-gRNA + 6 × 10^3^ Ad5-EF1α-EGFP. One well was used per group for each independent experiment, with a total of four independent experiments performed.

Five days later, cells from groups iii, iv, v, and vi were harvested and submitted to flow cytometry sorting (FACS) of green fluorescent protein-positive (GFP+) cells to remove any non-transduced cells (EGFP−), allowing a homogenous starting population for long-term culture (Supplemental Fig. 1B). GFP+ cells were plated on 24-well plates and cultured to 13 days post-infection (dpi) to allow cell division and dilution of episomes. An equal number of GFP− control cells from groups i and ii were also plated. Confluent monolayers at 13 dpi were collected to 6-well plates and cultured by splitting at: 17 dpi (split 1:2), 20 dpi (1:4), 27 dpi (1:4), and 33 dpi (1:2). Flow cytometry at 36 dpi determined the percentage of cells retaining GFP expression and sorted to select potentially integrated cells (Supplemental Fig. 1A). These terminally sorted cells were used for junction capture PCRs and quantitative PCR (qPCR) analysis of *EGFP* copy numbers, as well as *hexon* (the main adenoviral capsid protein gene) to enumerate the amount of remaining viral genomes.

### Flow cytometry

BNL-1NG cells were collected into sterile Hank's buffer B solution (Gibco, Grand Island, NY, USA) supplemented with 2% FBS and 1X streptomycin/penicillin (Sigma-Aldrich) and passed through 30 µm filter. Flow cytometry was performed on SONY SY3200 machines at the Siteman Flow Cytometry Core. GFP+ cells were sorted into DMEM/F12 media containing 25% FBS for one day to recover for use in the long-term culture experiments. Total flow results were obtained from four independent experiments.

### Targeted illumina deep sequencing

All DNA extractions were performed via phenol/chloroform extraction following incubation with RNaseA (GE Healthcare, Chicago, IL, USA), 10% sodium dodecyl sulfate (SDS) (Sigma-Aldrich) and proteinase K (Thermo Fisher Scientific, Waltham, MA, USA). DNA concentration was calculated with a value of absorbance of 1.0 at optical density (OD) 260 nm equivalent to 50 µg/mL of DNA. Whole genomic DNAs (gDNA) extracted from cells and organs were submitted to Illumina targeted deep sequencing via GEiC core services. PCR libraries (approximately 200 bp fragments) are generated using primers surrounding the target site, linkers are ligated, and Next Gen sequencing determines PCR sequences. Amplicon reads from fastq files are aligned using in-house script, based upon defined user-inputted start and end sequences. Individual alleles’ abundance are calculated as a percentage of total alleles present in each sample. DNA samples were submitted in duplicate with 15 µL of 100 ng/µL template.

### Junction capture PCR

*EGFP*-integrated *ROSA26* junction capture PCRs were performed with primers annealing within promoter EF1α (Supplementary Table 1, Oligo-1) or *EGFP* (Supplementary Table 1, Oligo-2) and genomic regions past homology sequences (Supplementary Table 1, Oligo-3 and Oligo-6). Twenty microliter of reactions contained 0.4 µL Phire II polymerase (Thermo Fisher Scientific), 0.5 µM dNTPS (New England Biolabs), 250 µM primers, 150 ng of template DNA (in vitro) or 250 ng (in vivo). In vitro and in vivo* EGFP*-integrated junction PCR reactions also contained primers (Supplementary Table, Oligo-4 and Oligo-5) to amplify a 616 bp viral sequence to confirm the presence of viral vectors and as a loading control. In vivo* hAAT*-integrated *ROSA26* junction capture PCR was performed with a primer located within the 3′ end of *hAAT* (Supplementary Table 1, Oligo-7) and a primer binding to the 3′ *ROSA26* locus, past the right homology sequence (Supplementary Table 1, Oligo-8). PCR gel images are representative of six independent PCRs and gels.

### Quantiative PCR

In vitro DNA copy number quantification was performed on a Light Cycler 480 (Roche, Basel, Switzerland) using 25 ng of purified DNA in 10 µL reactions. All samples were run in triplicate. qPCR reactions were performed with FastStart TaqMan Probe Master Mix according to the manufacturer's instructions (Roche). Standards were prepared as optically pure adenoviral plasmid DNA pAd5-EF1α-EGFP (*ROSA26* donor) tenfold serially diluted, containing template for *EGFP* (accession-C8CHS1) and *hexon* (accession-E1V2T2) genes. Sigma-Aldrich manufactured primer specificity was checked using BLAST alignment. *EGFP* copies were quantified with specific primers (Supplementary Table 1, Oligo-9 and Oligo-10) and a TaqMan probe (Supplementary Table 1, Oligo-11). *Hexon* copies were quantified with primers (Supplementary Table 1, Oligo-12 and Oligo-13) and a probe (Supplementary Table 1, Oligo-14). The quantification of *hexon* and *EGFP* genes was performed separately, with each individual reaction being normalized to the *m-Actin* gene (accession-A1E281) as a template loading control, using *m-Actin*-specific primers (Supplementary Table 1, Oligo-15 and Oligo-16) and probe (Supplementary Table 1, Oligo-17). Cycling conditions consisted of: initial denaturation (2 min at 95 °C), 45 cycles of amplification by 15 s at 95 °C, annealing 10 s at 58 °C, and elongation 45 s at 72 °C. Threshold cycle (*C*_t_) for each triplicate was determined by the point when fluorescence passed the threshold limit (tenfold the standard deviation of the baseline). Data analysis was performed using fit point method on Light Cycler 480 software (v1.5). Results are from two independent experiments, each containing two biological replicates; individual samples were run in triplicate.

DNA copy number quantification from in vivo experiments was performed on an Applied Biosystems StepOnePlus qPCR system using 50 ng of liver-extracted template DNA in 20 µL reactions. Standards for the *hAAT* and *hexon* genes were prepared by serial tenfold dilutions of optically pure adenoviral plasmid pAd5-EF1α-hAAT (*ROSA26* donor) containing both *hAAT* (accession-Q13747) and *hexon* targets. A commercial TaqMan Gene Expression assay (Hs00165475) (Applied Biosystems, Foster City, CA, USA) was used to determine *hAAT* copy numbers. *Hexon* gene copies were enumerated as described above. *Hexon* and *hAAT* quantification was performed separately, with individual reactions normalized to *m-Actin*. Cycling conditions and *C*_t_ calculations were performed as described above. Data analysis was performed using fit point method on StepOnePlus software. Results are from one animal experiment, with at least three biological replicates (two for negative control PBS group), and samples were run in quadruplicate qPCR reactions.

### Linear amplification-mediated qPCR

To determine integrations rates occurring in vivo mice were injected with PBS (*n* = 3), 1:1 mixture of Ad5-EF1α-EGFP to Ad5-gRNA (no nuclease, *n* = 6) or Ad5-EF1α-EGFP to Ad5-Cas9-gRNA (*n* = 6). Mice were sacrificed at 7, 21, and 42 dpi. The *EGFP* gene provides a permissible method for calculating large homology-directed repair (HDR)-mediated insertions, via linear amplification-mediated PCR (LAM-PCR) due to a common restriction site equidistant from the 3′ homology region in the transgene and genome. This method is used to avoid bias associated with PCR amplification of rare alleles.

LAM-PCR was performed similarly to previous reports, with 40× rounds of linear amplification from 100 to 800 ng of starting gDNA template [14, 23]. Fifty microliters of reactions used a biotin-conjugated primer binding downstream of *ROSA26* in reverse orientation (Supplementary Table 1, Oligo-18) and Phire II polymerase. Linear PCR products were bound to M280 streptavidin-conjugated magnetic beads (Invitrogen, Carlsbad, CA, USA) at 25 °C for 6 h. Second strand synthesis was performed with Klenow fragment (New England Biolabs) using random oligo-primers (Promega, Madison, WI, USA). Then, double-stranded DNA was restriction digested with *Nco*I (New England Biolabs) at approximately equal distances in both integrated and non-integrated alleles (2.01 and 1.99 kb, respectively) to generate equal-sized PCR fragments. *Nco*I sticky end-specific double-stranded linker (Supplementary Table 1, Oligo-19 and Oligo-20), containing nested PCR primer target sites, was annealed to digested PCR products using Fast-Link Ligase (Epicentre Technologies, Madison, WI, USA) or Quick Ligase (New England Biolabs). Linker was generated as described in detail elsewhere [14, 23]. Following ligation, two nested PCRs using two primers sets (first nested PCR: Supplementary Table 1, Oligo-21 and Oigo-22) (second nested PCR: Supplementary Table 1, Oligo-23 and Oligo-24) increased template quantity for qPCR.

Last, 25 ng of the second nested PCR was used in separate 10 µL qPCR reactions with unintegrated-specific (wild-type) primers and Taqman probe or integrated-specific (*EGFP*) primers and probe. Cycling conditions and threshold cycle estimation were performed as described above on a Roche LC480 machine. Data analysis was performed using fit point method. Samples were run in triplicate. Tenfold serially diluted plasmid DNA, containing *EGFP* (integrated *ROSA26* target) and *ROSA26* HAs (non-integrated wild-type allele target), served as copy number standard (Supplemental Fig. 1C, D). TaqMan primer and probe for *EGFP* are described above. Wild-type primers (Supplementary Table 1, Oligo-25 and Oligo-26) and probe (Supplementary Table 1, Oligo-27) are specific for the left *ROSA26* HA, absent from *Nco*I-digested *EGFP* LAM-PCR product. Results are from one or more independent LAM-PCR preps. Percentage of *EGFP*-integrated *ROSA26* alleles are calculated by dividing the number of *EGFP* copies by the total amount of copies present (*EGFP* copies/[wild-type copies + *EGFP* copies]), multiplied by 100.

### hAAT ELISA

Mice were injected with 10^11^ total VP mixture of a Ad5-EF1α-hAAT (donor) to Ad5-Cas9-gRNA mixture (1:1 donor: Cas9-gRNA [*n* = 6], or 3:1 donor: Cas9-gRNA [*n* = 5]), as well as a 1:3 mixture (*n* = 5). Concurrently, other mice received an equivalent amount of Ad5-EF1α-hAAT (donor) to Ad5-CMV-EGFP mixtures (sham) into mice at a ratio of 1:1 (*n* = 4) or 3:1 (*n* = 6) (donor: sham). Plasma was collected approximately biweekly.

hAAT concentrations in plasma were determined by a sandwich ELISA (enzyme-linked immunosorbent assay) assay (Supplemental Fig. 2A). Polyclonal goat anti-hAAT (4 µg/mL) (A80-122A, lot. 3 and 9) (Bethyl Laboratories Inc., Montgomery, TX, USA) antibodycoated MaxiSorp plates (Thermo Fisher Scientific) in coating buffer (sodium bicarbonate [pH 8.5]) at 4 °C. Recombinant hAAT was applied to each ELISA plate as standard concentrations ranging from 0.977 to 62.5 ng/mL (10306-H08H, lot. LCL06NO2242) (Sino Biologicals, Beijing, China). Diluent was 1X PBS, 0.05% Tween 20 (Sigma-Aldrich), and 0.5% bovine serum albumin (BSA) (Santa Cruz Biotechnology, Dallas, TX, USA). Results were from OD readings of diluted samples in the linear range of the assay (Supplemental Fig. 2B). No cross-reactivity to mouse plasma or BSA was observed (Supplemental Fig. 2C).

### Western blot

hAAT Western blots were performed using 2 µL of murine plasma diluted into 8 µL of 5X SDS Loading buffer (1 M Tris-HCl [pH 6.8], 10 g SDS, 50 mL glycerol, 25 mg bromophenol blue, 25 mL beta-mercaptoethanol, deionized H_2_O to 100 mL) and 30 µL of PBS. Half of the diluted samples or Spectra MultiColor BroadRange Marker (Thermo Fisher Scientific) was loaded after denaturation and ran in 10% TGX Mini Protean SDS-PAGE (sodium dodecyl sulfate-polyacrylamide gel electrophoresis) gels (Bio-Rad Laboratories, Hercules, CA, USA). Protein was transferred to PVDF membrane (Bio-Rad) after electrophoresis. Membranes were incubated 4 °C overnight with anti-tetra-His mouse IgG antibody (Qiagen, Hilden, Germany). Secondary antibody incubation with HRP-conjugated goat polyclonal anti-mouse IgG (Agilent) was followed by West Pico SuperSignal chemiluminescence detection (Thermo Fisher Scientific). Results shown are from four independent blots.

### Off-target site identification

Potential off-target sites of the *ROSA26* gRNA in the murine genome were predicted using two different computational approaches. First, in-house script from the GEiC was utilized with the most strict of conditions (>2 bp mismatch, up to 8 bp); second, the online tool GT-SCAN ([http://gt-scan.csiro.au/], Australia Bioinformatics Resource) was used with the highest max specificity (off-target = 2) and the 23 nt rule. Sites with the highest chance of gRNA targeting, based upon fewest base pair mismatches and distance from PAM site, were chosen from loci present in both computational analyses. Most chosen off-target sites contained 1 bp mismatch in the distal, non-seed, region of gRNA target sequence.

### Histopathology and vector toxicity analysis

Mice were submitted to the Pathology Core at the Department of Comparative Medicine at WUSTL for the following analyses: gross necropsy, liver weight, and liver histopathology, CBC white cell counts, and alanine transaminase/aspartate transaminase levels as markers of liver toxicity. Samples were submitted in a blinded manner and consisted of two negative control PBS-injected mice and three mice from integrative and non-integrative vector groups.

### Immunohistochemistry

At 7, 21, and 42 dpi mice were sacrificed for immunohistochemistry (IHC) staining of vector transgene expression. Tissue processing and section staining was performed as described elsewhere [24]. EFGP staining was performed with 1:1000 diluted primary chicken anti-GFP IgY antibody (Invitrogen), followed by incubation with secondary donkey IgG anti-chicken IgY conjugated to Alexa Fluor 488 (Jackson Immuno Research, West Grove, PA, USA), stained with SlowFade/DAPI (Invitrogen), and imaged on a confocal microscope.

### Statistics

ELISA data statistics was performed using parametric testing of individual time points between equivalent mouse groups. Statistical testing was completed using Real Stats Software (http://www.real-statistics.com/free-download/) suite in Microsoft Excel. An *F*test determined if variance between the two groups compared was significant or not, at each individual time point. If the *F*-test showed no significant difference in distribution, a non-paired Student's *t*-test#2 was used. If the *F*-test showed significant variance, a heteroskedastic *t*-test#3 was used as a conservative test of samples with unequal variance. All *t*-tests were analyzed by two-tailed. Additional Kruskal–Wallis testing, a non-parametric ranked statistical test, was used to transform non-normally distributed data (such as that of ELISAs) and validate significant differences between groups at individual time points. Animal numbers were chosen to allow ideal statistical power within technically feasible experimental analysis.

Statistics on qPCR data was performed using non-parametric unpaired *t*-test (two-tailed) of groups receiving equal amounts of initial donor vector and CRISPR/Cas9 or sham encoding vector. Significance testing was completed using Mann–Whitney *U* test on GraphPad StatMade software. Significance was based on *p*values with >0.05 being considered non-significant and <0.05 as significant.

## Results

### Adenovirus delivery of CRISPR/Cas9 results in knock-in of the *EGFP* gene at the *ROSA26* locus

**Fig. 2:**
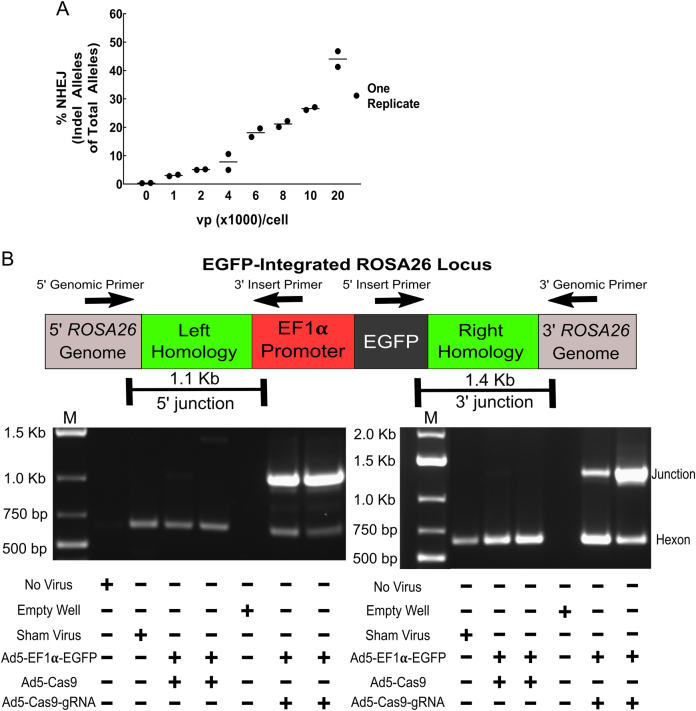
Targeted in vitro knock-in occurs following adenoviral delivery of CRISPR/Cas9. **a** Targeted Illumina deep sequencing of the *ROSA26* locus was performed on genomic DNA extracted from the BNL-1NG murine liver cell line following transduction with Ad5-Cas9-gRNA infection at various amounts of viral particles per cell (VP/cell), to determine insertion and deletion (indel) formation resulting from NHEJ DNA repair of DSBs. **b** BNL-1NG cells were transduced with either Ad5-EF1α-EGFP (donor) and Ad5-Cas9 (no gRNA) or Ad5.EF1α-EGFP (donor) and Ad5-Cas9-gRNA. Additional controls consisted of no virus and a sham Ad5 virus expressing an irrelevant coagulation factor gene driven by the CMV gene and lacking any CRISPR/Cas9 components. Genomic DNA was extracted for PCR amplification of integration junctions and 612 bp amplicon of viral *hexon* gene as a loading control to confirm the presence of viral vector transduction. Primer sets are composed of one primer within the genome (outside of the homology arms) and one primer within the donor transgene, to confirm on-target insertions. Junction PCR products were gel extracted and Sanger sequenced to confirm targeted integration (Supplemental Figs. 3 and 4). Gels are representative of four independent experiments with junction amplification being confirmed from early and late time points. Arrows show direction and position of PCR primers

To test if adenoviral delivery of CRISPR/Cas9 enables gene editing, a vector encoding Cas9 and gRNA (an artificial construct of *trans*-activating and CRISPR-associated RNA) targeted to murine “safe harbor” *ROSA26* locus was produced (Ad5-Cas9-gRNA) (Fig. 1). The murine liver cell line BNL-1NG was infected with this vector at various numbers of VPs per cell. Seventy-two hours later, cells were harvested and gDNA extracted. Targeted deep sequencing of *ROSA26* loci in the gDNA samples was performed to identify insertions and deletions (indels), indices of non-homologous end joining (NHEJ) DNA repair of double-stranded breaks (DSBs). Of note, increasing VP per cell resulted in increased indel formation at *ROSA26* (Fig. 2a). The most frequent mutations were small single base pair indels with larger indels occurring less frequently, while larger mutations were also detected (Supplemental Table 2A). This formation of indels demonstrated successful DSBs mediated by Ad5-Cas9-gRNA.

Next, we sought to explore if (i) HDR of DSBs could mediate transgene insertion at *ROSA26* and (ii) if such an insertion could provide long-term gene expression. To test these theories, we used co-infection of DSB-inducing Ad5-Cas9-gRNA and donor vector Ad5-EF1α-EGFP (containing an EGFP expression cassette with 0.8 kb of *ROSA26* homology sequences) (Fig. 1). BNL-1NG cells were co-infected with a 1:1 mixture of Ad5-Cas9-gRNA and donor vector Ad5-EF1α-EGFP, the “integrative group,” or equivalent amounts of Ad5-Cas9 (no gRNA) and Ad5-EF1α-EGFP as ectopic episomal expression controls, the “non-integrative group.”

First, to assay for targeted integration, a junction capture assay was performed on gDNA extracted from all viral-infected groups. PCR primers flanking the 5′ and 3′ homology sequences of the genomic *ROSA26* insertion site and primers within the promoter or the *EGFP* transgene were used to obviate any PCR products from episomal vector DNA or unintegrated loci. PCR products were produced in integrative groups receiving full CRISPR/Cas9 integration components, Ad5-Cas9-gRNA and Ad5-EF1α-EGFP (Fig. 2b). PCR product Sanger sequencing confirmed targeted genomic integration occurred (Supplemental Figs. 3 and 4).

Second, we utilized a long-term culture strategy to test episomal dilution’s versus targeted integration’s role in maintaining gene expression. Following a 36-day culture to dilute episomal vectors, we observed a non-significant increase in cells retaining EGFP expression (percentage GFP+) in integrative groups at two doses versus equivalent episomal expression controls (Supplemental Fig. 5A). EGFP positivity was gated on the PBS− control group, whereby all cells within this population were considered GFP− (Supplemental Fig. 1A). Non-integrative groups also retained some residual expression. Flow sorting of GFP+ cells was also performed to enrich for putative integrated cells. These GFP+ sorted cells from integrative and non-integrative groups were also used for junction capture PCR and determined HDR-mediated *EGFP* alleles were still present. Additionally, we used qPCR to investigate if *EGFP* copy numbers increased relative to ectopic control groups, due to cellular replication of integrated *EGFP*. In other words, we sought to enumerate remaining (non-replicative) episomal viral genomes within the cells and the *EGFP* transgene (potentially replicative via integration). The major Ad capsid protein gene, *hexon*, was used to quantify viral genomes. qPCR analysis detected viral genomes in both integrative and non-integrative groups, with a non-significant difference of 1.1 × 10^5^ copies/ng m-Actin (Supplemental Fig. 5B). Transgene copies (derived from both episomal and integrated genomic sources) showed a larger difference (difference of 6.4 × 10^6^ copies/ng m-Actin) between groups; however, this difference was also found to be non-significant (Supplemental Fig. 5B). Although in vitro dilution experiments did not generate a significant difference between integrative and episomal persistence of a reporter transgene, junction capture PCR determined integrated *ROSA26* loci could persist through a 36-day long-term culture.

### Somatic integration of the *hAAT* gene in mice provides long-term gene expression

**Fig. 3:**
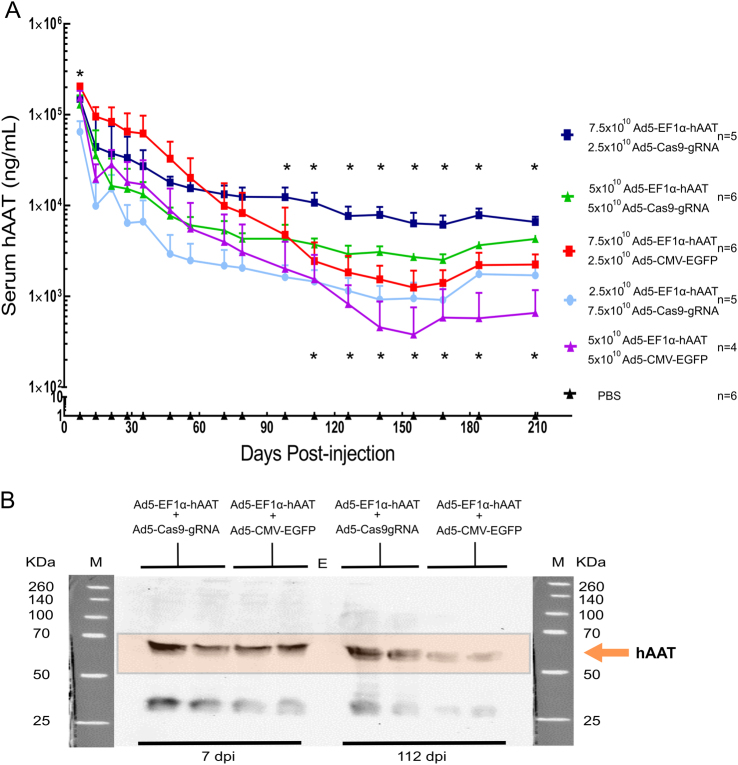
Somatic integration of hAAT maintains stable long-term gene expression in mice. **a** Mice were infected with a total of 1.0 × 10^11^ VP recombinant adenoviruses or PBS (negative control). Over a 210-day period, plasma levels of hAAT were determined intermittently via ELISA. Black triangles represent mice injected PBS (*n* = 6). Red squares and a line indicate mice (*n* = 6) injected with Ad5-EF1α-hAAT (7.5 × 10^10^ VP) and Ad5-CMV-EGFP (2.5 × 10^10^ VP). Purple triangles and a line represent mice (*n* = 4) injected with Ad5-EF1α-hAAT (5.0 × 10^10^ VP) and Ad5-CMV-EGFP (5.0 × 10^10^ VP). Dark blue squares and line indicate mice (*n* = 5) injected with Ad5-EF1α-hAAT (7.5 × 10^10^ VP) and Ad5-Cas9-gRNA (2.5 × 10^10^ VP). Green triangles and line show mice (*n* = 6) injected with Ad5-EF1α-hAAT (5.0 × 10^10^ VP) and Ad5-Cas9-gRNA (5.0 × 10^10^ VP). Closed circles and line with a light blue color indicate a mice group (*n* = 5) injected with Ad5-EF1α-hAAT (2.5 × 10^10^ VP) and Ad5-Cas9-gRNA (7.5 × 10^10^ VP). *Significant differences with a *p*value < 0.05 between equivalent groups, while an absence of asterisk represents no significant difference. The top asterisk row represents the 3:1 vector group comparison (Ad5-EF1α-hAAT: Ad5-CMV-EGFP or Ad5-EF1α-hAAT: Ad5-Cas9-gRNA) (squares) and the bottom asterisk row represents 1:1 vector groups (triangles). Error bars are s.d. of the mean. **b** One microliter of plasma samples were run on SDS-PAGE followed by Western blot analysis to visualize the temporal change of gene expression between day 7 and day 112 in integrative versus episomal groups. Each lane represents one sample from one mouse. Image is representative of three blots. M and kDa indicate molecular mass marker and kilo-Daltons, respectively

To determine if cDNA knock-in could provide long-term expression of a therapeutic serum protein, we used a hAAT expression model in mice. Various ratios of Ad5-EF1α-hAAT (donor) to Ad5-Cas9-gRNA (Cas9-gRNA/integrative) or an equivalent amount of Ad5-EF1α-hAAT (donor) to Ad5-CMV-EGFP (sham/non-integrative) mixtures were injected into mice (10^11^ total VP for all mice). The use of Ad5-CMV-EGFP, a classical control vector, ensured uniform amount of total virus administered to mice. Plasma was collected to determine hAAT levels using a human-specific ELISA. Initial hAAT levels (peaking at approximately 250 µg/mL) at 7 dpi showed non-significant differences between equivalent groups, with the exception of the 3:1 equivalent groups in which non-integrative mice had significantly higher hAAT levels. No other significant differences were seen until approximately 100 dpi, when non-integrative groups’ (donor + sham) hAAT levels decreased to levels below their integrative counterparts (donor + Cas9-gRNA). This trend continued to a point whereby 3:1 and 1:1 ratios of Ad5-EF1α-hAAT to Ad5-Cas9-gRNA (donor to Cas9-gRNA) administered mice had significantly higher gene expression than any non-integrative groups (Fig. 3a). By 210 dpi, mice receiving the integration system (donor to Cas9-gRNA) had 6.5-fold (1:1 ratio groups) and 2.9-fold (3:1 ratio groups) greater levels of hAAT expression versus their non-integrating counterparts (donor to sham), even though mice had received equivalent amounts of total and hAAT-expressing virus (Fig. 3a). Furthermore, the 1:3 ratio of Ad5-EF1α-hAAT to Ad5-Cas9-gRNA group, receiving the least amount of hAAT-encoding vector of any group, maintained nearly equal or higher levels of hAAT than any non-integrative group, including positive control mice receiving 1 × 10^11^ VP of adenovirus vector expressing hAAT from the CMV promoter (Supplemental Fig. 6). Western blot analysis of multiple plasma samples from 112 dpi showed stronger AAT detection among integrative mice relative to equivalent non-integrative mice samples, supporting ELISA results (Fig. 3b). Taken together, these data showed *hAAT* cDNA knock-in achieved long-term gene expression lasting over 200 days.

### Somatic integration of *hAAT* gene occurred at the target *ROSA26* locus and contributed to the increased *hAAT* gene maintenance over time

**Fig. 4:**
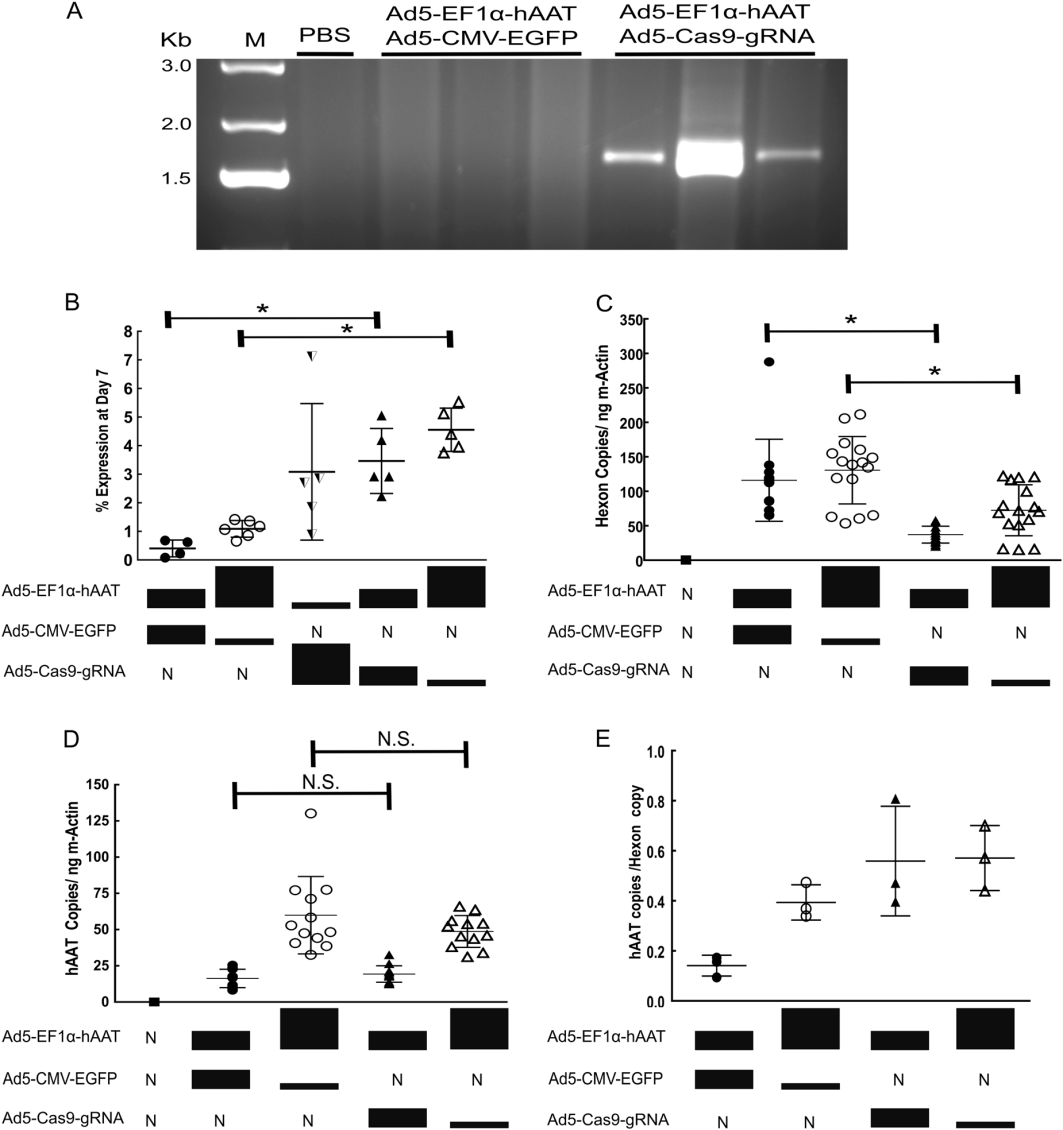
Targeted gene integration contributes to persistence of gene expression. **a** A 3′ junction capture PCR of genomic DNA was performed to determine the presence of *hAAT*-integrated *ROSA26* alleles (expected size, 1.7 kb) at 210 days post-injection. Each lane represents amplicons from an individual mouse’s genomic DNA from groups in Fig. 3a. **b** Serum levels of hAAT at 210 days were compared to initial serum levels at day 7 for individual mice. Each data point represents one mouse. *Significance with *p*value <0.05 (*p* = 0.0159 and 0.0043, respectively). “N” denotes none. **c** Liver-extracted DNA was used as a template to quantify *hexon* to determine the amount of residual viral episomes (*p* = < 0.0001 and *p* = 0.0017, respectively). Each dot represents one qPCR replicate with four replicates per mouse. **d***hAAT* copy numbers remaining at 210 dpi were quantified by qPCR (*p* = 0.1957 and 0.3724, respectively). Each dot represents one qPCR replicate with four replicates per mouse. NS represents non-significance with *p*value >0.05. **e** The amount of *hAAT* copies per *hexon* copy present is shown per group. Each dot represents one mouse, with three mice per group. Error bars are s.d. of the mean. Closed circles represent mice injected with Ad5-EF1α-hAAT (5.0 × 10^10^ VP) and Ad5-CMV-EGFP (5.0 × 10^10^ VP). Open circles are mice injected with Ad5-EF1α-hAAT (7.5 × 10^10^ VP) and Ad5-CMV-EGFP (2.5 × 10^10^ VP). Half closed, downwards triangles represent mice injected with Ad5-EF1α-hAAT (2.5 × 10^10^ VP) and Ad5-Cas9-gRNA (7.5 × 10^10^ VP). Closed triangles are mice injected with Ad5-EF1α-hAAT (5.0 × 10^10^ VP) and Ad5-Cas9-gRNA (5.0 × 10^10^ VP). Open triangles represent mice injected with Ad5-EF1α-hAAT (7.5 × 10^10^ VP) and Ad5-Cas9-gRNA (2.5 × 10^10^ VP)

To validate *hAAT* integration at *ROSA26*, junction capture PCR of liver genomic DNA was performed. Mice receiving Ad5-EF1α-hAAT and Ad5-Cas9-gRNA showed PCR amplification of 3′ integrated genomic junctions, while control mice (Ad5-EF1α-hAAT and Ad5-CMV-EGFP) showed no PCR product (Fig. 4a). PCR amplification of 5′ junctions from in vivo was unsuccessfully imaged due to strong secondary structures as this junction contains long GC repeat tracks, which are known to inhibit PCR amplification (Supplemental Fig. 3B) [25]. At 210 dpi residual hAAT serum levels from integration groups was between 2.2 to 5.5% (averaging 4%) of their initial expression level at week 1, while our non-integrating episomal expression groups showed between 0.08 to 1.4% (average 0.8%) of their initial week 1 expression (Fig. 4b). To quantify the presence of remaining virus, viral genomes from DNA samples extracted from liver tissue were counted using qPCR. Non-integrative groups (donor to sham) showed the highest quantity of vector genomes remaining (Fig. 4c). Integrative groups showed significantly less viral genomes. However, equal copy numbers of *hAAT* were detected between groups (Fig. 4d), resulting in a higher ratio of *hAAT* copies present per *hexon* copy present in integrative groups than seen in non-integrative groups (Fig. 4e). Non-integrative mice lost less virus genomes than equivalent integrative counterparts, but integrative groups maintained similar numbers of *hAAT* copies despite the greater loss of virus. Thus, the maintenance of *hAAT* copies likely occurred due to integration of *hAAT* at *ROSA26*, whereby integrated *hAAT* copies would be passed down to both daughter cells of a proliferating cell. If integration did not influence *hAAT* genetic persistence, non-integrated *hAAT* copies would be expected to dilute and be lost at a rate similar to the observed loss of viral genomes. We also hypothesize that the presence of Cas9, a large bacterial nuclease, may have contributed to the greater loss of virus in integrative groups. However, this loss of vector was compensated by the maintenance of *hAAT* copies through targeted integration. Random integration was not thought to contribute to the persistence of *hAAT* copies because if whole viral genomes integrated, both groups could be expected to have similar remaining *hexon* copy numbers. Together, evidence of targeted integration at *ROSA26* and the maintenance of *hAAT* alleles despite a significant loss of virus in integrative groups confirms integration’s contribution to the long-term expression of the serum protein.

### Adenoviral-mediated delivery and knock-in of the *EGFP* gene at *ROSA26* shows persistent gene expression occurs in both integrated and non-integrated mice

**Fig. 5:**
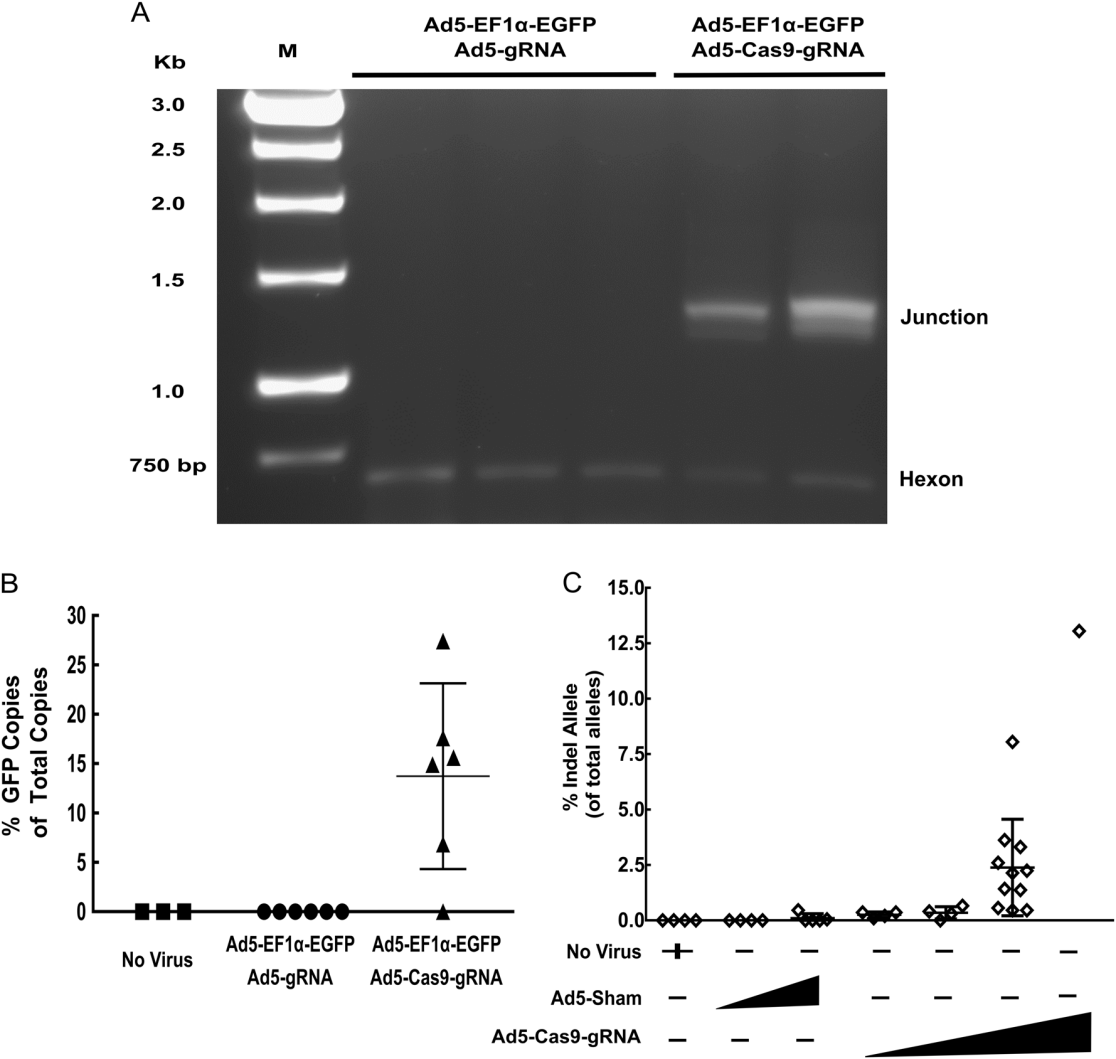
Knock-in and NHEJ rates following in vivo delivery with adenovirus is variable and persists over time. A total of 1 × 10^11^ viral particles of Ad5-EF1α-EGFP and Ad5-Cas9-gRNA (*n* = 6), or Ad5-EF1α-EGFP and sham Ad5-gRNA (*n* = 6), were injected into mice at a ratio of 1:1. PBS mock injected mice served as a no virus control (*n* = 3). **a** Junction capture PCR was used to amplify 3′ junctions from *EGFP-*integrated *ROSA26* alleles from genomic DNA from the mice. Each lane represents genomic DNA template from one mouse. Viral amplification of 612 bp of the *hexon* gene served as a loading control and confirmed viral transduction. **b** LAM-PCR was performed by production of single-stranded linear PCR product from a biotinlyated primer binding downstream of the 3′ homology region of *ROSA26* in the mouse genome, whereby both integrated and non-integrated alleles are amplified. qPCR analysis to quantify HDR-mediated integration rates is displayed as percent *EGFP-*integrated alleles out of total *ROSA26* alleles. Error bars are s.d. of the mean. **c** Mice injected with Ad5-Cas9-gRNA or a sham virus were sacrificed at time points ranging between 7 and 210 days and genomic DNA was extracted. This DNA, and genomic DNA extracted at the conclusion of the hAAT experiment in Figs. 3 and 4, was used as template for targeted Illumina deep sequencing of the *ROSA26* for the presence of edited alleles, witnessed by NHEJ-induced indels

To quantify gene editing rates, a second in vivo experiment was performed. Mice were injected with either PBS, Ad5-EF1α-EGFP (donor) and Ad5-Cas9-gRNA (CRISPR), or Ad5-EF1α-EGFP (donor) and Ad5-gRNA (no nuclease, non-integrative). The *EGFP* gene allows calculation of large insertion editing rates via LAM-PCR (see Methods). To confirm the EGFP donor vector could also facilitate integration in vivo following induction of DSB, liver gDNA was used as template for junction capture as described above. Mice receiving Ad5-EF1α-EGFP and Ad5-Cas9-gRNA showed *EGFP* cDNA integration at the *ROSA26* locus (Fig. 5a). No integrated alleles in mice receiving the *EGFP* donor vector and gRNA vector (no nuclease) were detected, supporting the idea that integration did not occur at *ROSA26* in the absence of DSB. To qualitatively explore transduction, IHC staining of EGFP in the liver tissue of mice was performed, showing extensive viral transduction in all virus-injected animals and persistent gene expression from all groups (Supplemental Figure 7). Next, HDR-mediated insertion rates at *ROSA26* in mice receiving Ad5-EF1α-EGFP (donor) was quantified using LAM-PCR. Calculated integration rates ranged between approximately 5.0 and 26% of the total detected *ROSA26* alleles in five of the six mice receiving Ad5-Cas9-gRNA in conjunction with the *EGFP* donor (Fig. 5b). Estimation of the number of liver cells harboring *EGFP*-integrated *ROSA26* is likely to be dependent on whether integration events were mono-allelic, bi-allelic, or both. For example, if all integration events were bi-allelic, then integration rates would correlate to 2.5 to 13% of total liver cells harboring *EGFP*-integrated *ROSA26* loci. Importantly, the one integrative mouse that failed to display integrated junctions also lacked detectable integration via linear amplification-mediated qPCR (LAM-qPCR).

Next, liver gDNA extracted from livers of mice receiving Ad5-Cas9-gRNA from both in vivo experiments was submitted to deep sequencing of *ROSA26* for quantification of NHEJ gene editing events. Comparing vector doses showed that the editing activity was dependent on vector dosage (Fig. 5c). Indel formation favored smaller mutations, similar to in vitro findings (Supplemental Table 2B). When an equal dose of Ad5-Cas9-gRNA was graphed by time since injection, a general decrease in abundance of genetically altered *ROSA26* alleles was observed. Yet, genetically altered alleles were still detectable over 200 days post-injection (Supplemental Fig. 8). Detection of NHEJ gene editing in other organs of a mouse was explored and found to be majorly in the liver of the animal (Supplemental Figure 9).

### Vector toxicity and off-target analysis show no overt long-term consequences of gene editing

**Fig. 6:**
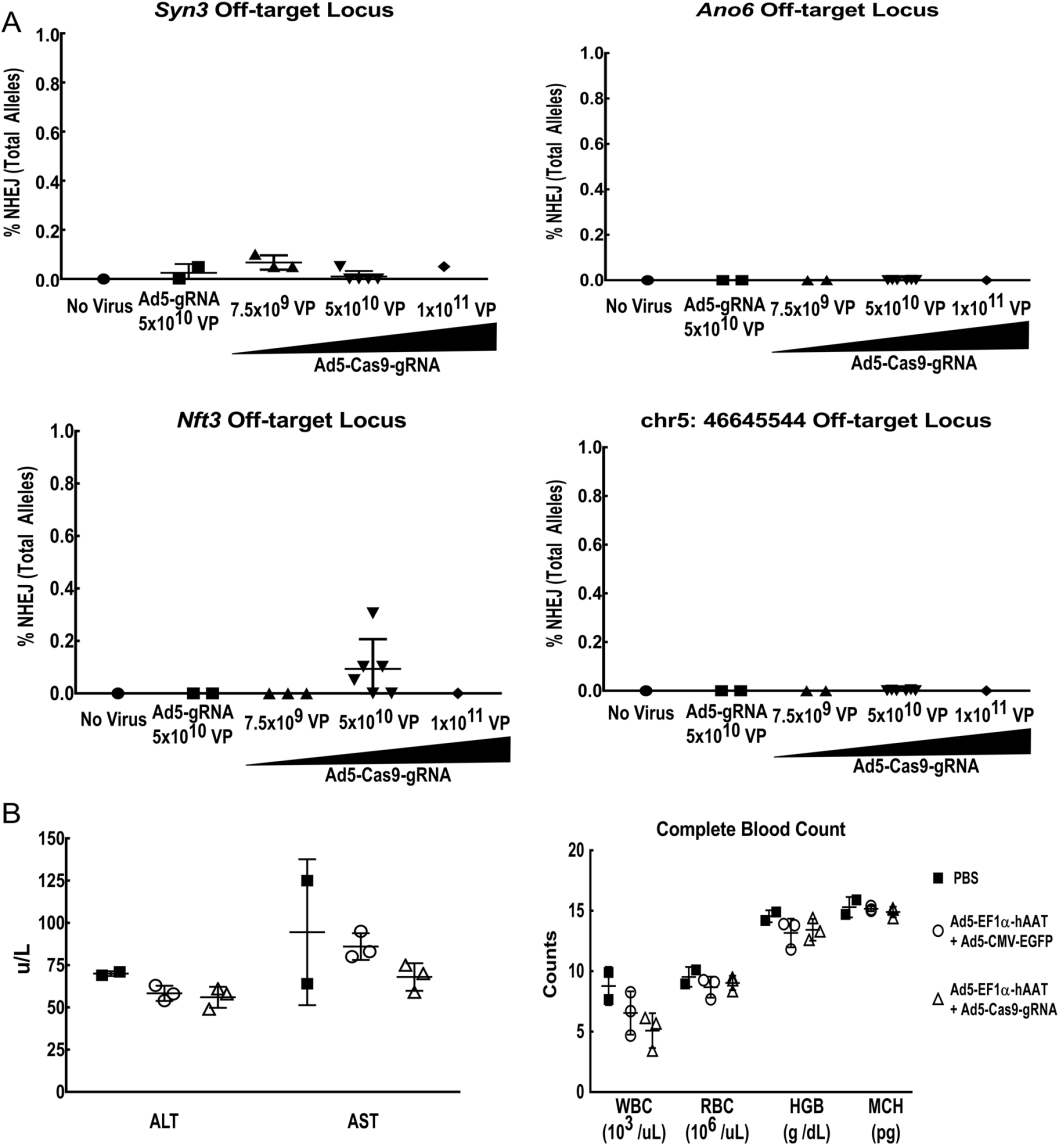
Off-target analysis and vector toxicity show little long-term consequences of gene knock-in. **a** Computationally derived top off-target candidate sites (*Syn3*, *Ano6*, *Nft3*, and chr5: 46645544) were submitted to targeted Illumina deep sequencing for evidence of NHEJ activity based upon indel formation following imperfect DNA repair. Amount of virus administered had no effect on the presence of indel formation at the selected candidate off-target sites. Each dot represents data from one individual mouse. **b** Mice were submitted in a blinded manner to gross pathology examination with an emphasis on liver toxicity markers (ALT/AST transaminases) and whole blood cell counts levels, 7 months after receiving hAAT-encoding vectors. Each dot represents data from one mouse. Error bars are s.d. of the mean. hAAT donor is Ad5-EF1α-hAAT and GFP sham is Ad5-CMV-EGFP. WBC white blood count, RBC red blood count, HGB hemoglobin, MCH mean cell hemoglobin

Last, we sought to determine whether in vivo gene editing resulted in unintended editing within the mouse genome. The top four computationally derived off-target candidate loci were submitted to targeted deep sequencing. Results showed little to no detectable off-target gene editing at the four chosen loci regardless of vector dose (Fig. 6a). Additionally, we sought to explore long-term consequences of virus-mediated gene editing. Mice from all groups in the hAAT long-term gene expression experiment were analyzed for pathology and indices of liver toxicity by trained pathologist 200 dpi. All mice appeared in overall good health and no gross lesions were seen in any mice, although one virally treated mouse had a dilated left ventricle in the heart without overall cardiomegaly (Supplemental Fig. 10A, B). Histopathology of livers showed no abnormal pathology and only normal signs of aging including vacuolation of hepatocytes and hematopoiesis, including mice receiving PBS-only (no virus) injections. Indices of liver toxicity, transaminase levels, were normal in all mice and complete blood counts showed no noteworthy differences between groups (Fig. 6b, Supplemental Fig. 10C, D).

## Discussion

In this study, we employed adenovirus to achieve in vivo gene transfer at efficiency levels which feasibilized gene editing-based knock-in of corrective alleles. The ability of the delivered CRISPR/Cas9 system to achieve a targeted knock-in at a defined genomic locus potentially provides a strategy to circumvent genotoxic risk associated with integrating vectors. The efficiency of the process was fostered by the unparalleled in vivo gene delivery capacity of adenovirus. On this basis, knock-in was achieved at levels allowing serum augmentation based on functional expression of the corrective allele. Of note, the combination of CRISPR/Cas9 with adenovirus now provides a novel platform that potentially accrues the advantages of adenovirus, in combination with long-term gene expression.

First, successful gene transfer of the CRISPR/Cas9 system was confirmed by in vitro evidence of NHEJ repair of DSBs (Fig. 2a). High-efficiency gene editing was achieved at higher doses, approximately 50%. We would note that this high-efficiency in vitro editing was at a short time period after transduction. Next, in vitro co-transduction with a donor vector containing homology sequences confirmed that targeted insertion was possible at the *ROSA26* locus via HDR of DSBs (Fig. 2b). By modeling cellular division through a long-term culture scheme, we observed that cells were able to retain *EGFP-*integrated *ROSA26* alleles over the 36-day time course (Fig. 2b). However, *EGFP* cDNA copy numbers were not significant different between integrative and non-integrative groups in long-term culture, potentially due to a lack of selectable marker and the arbitrary selection imposed by random culture splits during each passaging. In essence, cells with *EGFP*-integrated alleles that would retain EGFP expression could have been lost unevenly during any of the four splits during culturing. Overall, our in vitro model of episomal dilution and cellular proliferation suggested that genetically altered alleles could persist over time in vitro. Interestingly, viral genomes were detected in both integrative and non-integrative groups even after 36 days of culturing, likely the result of residual episomal virus persisting through passaging. Adenoviral vectors have been well documented to persist both in vitro and in vivo [26–28].

Next, we used targeted *hAAT* knock-in to explore long-term gene expression using adenoviral vectors in an immune-competent model, as cellular turnover and episomal dilution could proceed differently in vivo. Our study found that serum protein levels lasted the duration of the experiment, approximately 200 days, in both integrative and non-integrative mouse groups (Fig. 3a). This study represents the first in vivo hAAT full cDNA knock-in and the longest tracking of somatic gene editing using adenoviral delivery of CRISPR/Cas9. Importantly, hAAT expression in all mice receiving episomal non-integrative vectors fell to a residual level of approximately 2.2 µg/mL or less, while integration-capable groups averaged 2.9-fold and 6.5-fold greater levels of hAAT in serum, representing a marked divergence from their non-integrative counterparts (Fig. 3). By the end of the time course, mice receiving the integrative system had serum levels nearly equivalent to or greater than the highest expressing non-integrative mice, even when as little as one-third hAAT-encoding virus was administered. Thus, in vivo somatic knock-in appears to be an effective approach to maintaining expression of a serum protein. Interestingly, the group maintaining the highest hAAT serum levels received three parts *hAAT* donor vector to one part CRISPR/Cas9-encoding vector. This trend suggests the importance of multiple copies of repair template to achieve efficient integration, as seen in another integration study [11].

The presence of *ROSA26 hAAT-*integrated alleles remaining at the end of the 200-day experiment was confirmed by junction amplification PCR from liver-extracted gDNA (Fig. 4a). The role of integration in maintaining long-term gene expression was further evidenced by qPCR data (Fig. 4b). Between integrative and non-integrative groups, there was a significant reduction in remaining adenoviral genomes in integrative groups relative to equivalent non-integrative groups (Fig. 4c). We believe that the increased loss of vector genomes in mice receiving the integrating system, compared to control treated mice, is potentially related to immunogenicity and cytotoxicity of the large bacterial Cas9 nuclease protein [29, 30]. However, *hAAT* copy numbers were not significantly different between the two groups (Fig. 4d). These results show that for the same amount of DNA present, there were the same amount of *hAAT* copies in both groups, but more adenoviral vector genomes in non-integrative mice. As the integrative groups lost more viral genomes, the compensatory increase in *hAAT* copies (in integrative groups) is indicative of integrated transgene copy replication during cellular proliferation. If substantial integration did not occur, then *hAAT* copies would be significantly reduced in integrative groups similar to the observed significant reduction in remaining viral genomes. Although random integration could influence *hAAT* copy numbers, it is thought to be unlikely due to Ad5’s natural resistance to spontaneous integration which has been calculated to be approximately 6.72 × 10^−5^ heterologous integration events per transduced hepatocyte [31]. Thus, persistent gene expression in non-integrative groups was likely due to residual episomal persistence, a well-documented characteristic of Ad5 vectors [26–28]. This theory is supported by the high degree of similarity between our residual *hAAT* levels in episomal controls and published values [27, 28]. Additionally, recent studies have shown adenoviral vectors can achieve long-term gene expression in certain contexts [32–34]. Furthermore, a simultaneous positive control experiment using an adenovirus expressing *hAAT* under the control of the CMV promoter, lacking any integrative components, maintained some residual gene expression over the 210 day length of our experiment (Supplemental Fig. 6). IHC staining of murine livers at 7, 21, and 42 dpi also displayed continued gene expression in both integrating and non-integrating vectors, supporting the data seen in our in vitro model, the hAAT ELISA, and qPCR assays (Supplemental Fig. 7). Contribution of other organs to extended gene expression was likely minimal as we observed the most appreciable gene editing in the liver of the mice (Supplemental Fig. 9). However, we would note that while HDR repair results on-target integration, it is also possible that other DNA repair mechanisms can result in on-target integration [35, 36]. Taken together, these results are indicative of integrated transgene copy replication with simultaneous loss of vector genomes, from cell division as the juvenile mice aged or cellular proliferation that can occur following high doses of adenovirus vectors [37]. These findings also suggest that integrated genomic gene expression is potentially more stable or more robust than residual episomal-based expression. In this scenario, the gene expression differences despite equal *hAAT* copy numbers could be explained by potential transcriptional differences between the ubiquitously expressed and silencing-resistant *ROSA26* locus and an episomal environment [38–40]. The well-documented silencing of vector-based gene expression is also a possible explanation of gene expression differences despite the equal *hAAT* copies [41–43]. The last explanation would be the difference in expression is an artifact of the ELISA analysis; however, this explanation is thought to be unlikely, as all integrative mice had significantly greater expression than the one-to-one non-integrative group, even integrative mice receiving half the amount of hAAT-expressing virus. Furthermore, non-integrative controls groups received equal amounts of total virus and theoretically should persist similarly to the integrative groups, should integration not be a major driver of expression persistence. Thus, increased stable expression levels of the serum protein in integrative groups was likely a result of the prolonged persistence of genetically edited alleles.

In this study, we show that the level of integration occurring with adenoviral vector-mediated gene editing is high enough to effect the temporal expression of a transgene. Quantification of in vivo knock-in rates at *ROSA26* using LAM-PCR and an *EGFP* cDNA donor averaged 13.7% of total *ROSA26* alleles from genomic liver DNA (Fig. 5). Quantification of NHEJ mediated by CRISPR/Cas9 activity ranged from approximately 0.5%, at the lowest doses or later time points, to 13.5%. We noted that the in vivo NHEJ quantification in Fig. 5c is from samples harvested between 7 and 200 dpi, while in vitro NHEJ quantification in Fig. 2a is from 3 dpi. The amount of time since transduction could potentially effect editing rates if cells are lost due to cellular turnover, toxicity, or immune clearance. Overall, the rate of NHEJ detected in vivo suggests high transduction, similar to approximately 500 to 1000 VP per cell in vitro. The robust HDR rates are potentially a result of the immature age of the mice and adenovirus’ reported ability to inhibit the NHEJ pathway leading to a compensatory increase in HDR activity [44–46]. Additionally, our high transduction coupled with sizable donor HAs, an active gRNA, permissibility of integration at *ROSA26*, and induction of cellular cycling due to vector-mediated toxicity may have also contributed to the high insertional rate, as previously reported [47–49] (Supplemental Figure 7). Furthermore, larger indel formations not detectable by targeted deep sequencing may occur, potentially limiting detection of NHEJ activity [50].

Importantly, we found little off-target editing at four computationally derived top off-target candidate loci, even at the highest vector dose (Fig. 6). Although a recent controversial report identified unexpected mutations occurring upon CRISPR/Cas9 editing, it is beyond the scope of our project to identify all potential genome-wide off-target effects [51]. We also note this study identifying unexpected editing consequences was performed with embryonic injection, in the context of a developing organism with highly variable DNA repair activity and cellular differentiation. Conversely, our approach is based upon somatic editing of limited organ tissues. Although we cannot rule out damage-induced cellular proliferation as a contributor to persistent gene expression, no elevated transaminases were detected at the conclusion of the experiment (Fig. 6). This data showed that any vector-associated liver toxicity was absent by this later time point (Fig. 6). Early transaminase level spikes are well-documented with adenoviral gene delivery [52]. However, we measured ALT/AST at a later time point to assay for the presence of long-term liver damage, consistent with our focus on long-term outcomes of in vivo gene editing. Additionally, late time point increases in transaminase levels have also been documented with viral vectors [10]. Importantly, necropsy showed no lesions, malignancies, or other major signs of adverse health consequences in the animals receiving integrative vector treatment (Fig. 6, Supplemental Fig. 10).

Until now, it has remained unclear whether adenovirus can accomplish relevant lasting gene expression of a serum protein using CRISPR/Cas9. In this regard, most knock-in strategies have been largely based upon AAV vectors, showing much promise for conditions in which rare editing events are sufficient to significantly impact a disease phenotype [11–13]. However, AAV-based strategies are cautioned by increasing reports of unintended and potentially hazardous off-target integration events [53–58]. Despite a well-characterized immune response to adenoviral vectors [59] and a likely in vivo immune response against Cas9 per se [60], our work showed that knock-in gene editing via adenoviral vector is efficient enough to persist over time and did not have overt detrimental effects on the animals' health. Our results, taken with an overall lack of mortality in the study, allow the conclusion that adenovirus vector-mediated knock-in was well tolerated in the animals, contrary to a previous report of adenovirus as unsuitable for therapeutic gene editing [12]. Yet, for such a strategy to be applicable to humans, a safe harbor must be identified and thoroughly characterized; this endeavor is an area of active study with several genomic locales being proposed [61, 62].

Recently, in situ gene editing and knock-in at a safe locus have become promising strategies for long-term correction of inherited diseases. Our study, the first to report in vivo knock-in of several kilobases of an expression cassette encoding a serum protein at *ROSA26*, highlights that the gene editing approach is equally important as the vector used. For example, we target a neutral locus to avoid detrimental NHEJ-induced mutations, as this unintended consequence can limit the therapeutic index of in situ correction of an endogenous gene [63]. The gene editing study asserting adenovirus toxicity as the cause of no therapeutic gains successfully accomplished about 5.0% HDR-mediated correction of a disease allele. However, a 25% induction of detrimental NHEJ mutations at the endogenous locus likely abolished phenotypic correction from HDR [12]. Additionally, strategies targeting a safe harbor locus for full cDNA knock-in could be more commercially and clinically feasible with the ability to treat patients regardless of causative mutation. Such a strategy could be applied to many serum deficiencies, including AAT deficiency, as numerous causative mutations with varying disease phenotypes have been characterized [64]. Conversely, in situ gene correction of a specific mutations may be more efficacious or necessary for very rare disorders.

Moreover, adenoviral-mediated gene editing may have other benefits including fidelity as a donor template, a large packaging capacity (up to 35 kb with gutless Ads) for knock-in of large genes, and modifiable tropism [65, 66]. Many studies, including ours, target the liver as it is an optimal organ for corrective therapy of serum deficiencies. Although the use of adenoviral vectors for liver-directed gene therapy of genetic disorders is not suitable due to adenovirus’ toxicity, retargeting to different cell types or organ tissues may allow increased systemic transduction, thus increasing the number of cells that can be genetically altered while avoiding liver toxicity. In this regard, targeted vectors to specific cell types or tissues will potentially be powerful tools for efforts to correct some serum protein deficiency diseases, especially those that are not hepatocyte-sourced [67, 68]. For example, transduction of lung endothelium for AAT production may provide increased therapeutic benefits for patients with lung pathology at lower systemic levels of gene expression than those achieved in this study, via local expression in the disease-afflicted tissue. Furthermore, the use of stronger promoters than EF1α or optimized expression cassettes could also result in greater AAT expression [69]. Future gene therapies for serum deficiencies could also benefit from the use of hyper-active proteins or motifs that prolong protein in vivo stability [70, 71].

Although we did not seek to treat a disease state in this study, we show a generalizable knock-in at a specific genomic location can achieve lasting gene expression by altering a non-integrative vector into a system capable of site-specific integration. Approaches such as ours may be a feasible method to overcome issues associated with random integration, as is the nature of some vectors. Next, we will seek to determine if the ability of gene editing to persist in vivo and maintain stable levels of a serum factor can phenotypically alter a disease state in future studies. Additionally, further studies will be necessary to fully characterize immune responses to both CRISPR/Cas9 and the vectors which deliver it. These potential immunological and genotoxic consequences of gene editing using viral vectors will require exhaustive studies before clinical use should be advanced. Ultimately, our data support the development of viral-based gene editing strategies as a feasible approach to providing lasting therapeutic gene expression of serum proteins.

### Availability of data and material

The datasets supporting the conclusions of this article are included within the article and its additional files.

## Electronic supplementary material

Supplemental Table 1(PDF 280 kb)

Supplemental Table 2(PDF 51 kb)

Supplemental Figure Legends(DOCX 15 kb)

Supplemental Figure 1(TIF 12508 kb)

Supplemental Figure 2(TIF 3245 kb)

Supplemental Figure 3 A(TIF 17052 kb)

Supplemental Figure 3 B(TIF 6613 kb)

Supplemental Figure 4 A(TIF 16140 kb)

Supplemental Figure 4 B(TIF 16274 kb)

Supplemental Figure 5(TIF 2768 kb)

Supplemental Figure 6(TIF 2490 kb)

Supplemental Figure 7(TIF 37101 kb)

Supplemental Figure 8(TIF 2287 kb)

Supplemental Figure 9(TIF 2337 kb)

Supplemental Figure 10(TIF 3880 kb)

## References

[1] Vanden-Driessche T, Vanslembrouck V, Goovaerts I, Zwinnen H, Vanderhaeghen ML, Collen D (1999). Long-term expression of human coagulation factor VIII and correction of hemophilia A after in vivo retroviral gene transfer in factor VIII-deficient mice. Proc Natl Acad Sci USA.

[2] Tran R, Myers DR, Denning G, Shields JE, Lytle AM, Alrowais H (2017). Microfluidic transduction harnesses mass transport principles to enhance gene transfer efficiency. Mol Ther.

[3] Hu P, Li Y, Sands MS, McCown T, Kafri T (2015). Generation of a stable packaging cell line producing high-titer PPT-deleted integration-deficient lentiviral vectors. Mol Ther Methods Clin Dev.

[4] Cesana D, Ranzani M, Volpin M, Bartholomae C, Duros C, Artus A (2014). Uncovering and dissecting the genotoxicity of self-inactivating lentiviral vectors in vivo. Mol Ther.

[5] Braun CJ, Boztug K, Paruzynski A, Witzel M, Schwarzer A, Rothe M (2014). Gene therapy for Wiskott-Aldrich syndrome—long-term efficacy and genotoxicity. Sci Transl Med.

[6] Smith RH (2008). Adeno-associated virus integration: virus versus vector. Gene Therapy.

[7] Penaud-Budloo M, Le Guiner C, Nowrouzi A, Toromanoff A, Chérel Y, Chenuaud P (2008). Adeno-associated virus vector genomes persist as episomal chromatin in primate muscle. J Virol.

[8] Nathwani AC, Reiss UM, Tuddenham EG, Rosales C, Chowdary P, McIntosh J (2014). Long-term safety and efficacy of factor IX gene therapy in hemophilia B. N Engl J Med.

[9] Manno CS, Pierce GF, Arruda VR, Glader B, Ragni M, Rasko JJ (2006). Successful transduction of liver in hemophilia by AAV-Factor IX and limitations imposed by the host immune response. Nat Med.

[10] Ertl HC, High KA (2017). Impact of AAV capsid-specific T-cell responses on design and outcome of clinical gene transfer trials with recombinant adeno-associated viral vectors: An evolving controversy. Hum Gene Ther.

[11] Li H, Haurigot V, Doyon Y, Li T, Wong SY, Bhagwat AS (2011). In vivo genome editing restores hemostasis in a mouse model of hemophilia. Nature.

[12] Guan Y, Ma Y, Li Q, Sun Z, Ma L, Wu L (2016). CRISPR/Cas9‐mediated somatic correction of a novel coagulator factor IX gene mutation ameliorates hemophilia in mouse. EMBO Mol Med.

[13] Wang L, Yang Y, White J, McMenamin D, Bell P, Wilson JM (2016). CRISPR/Cas9-mediated in vivo gene targeting corrects haemostasis in newborn and adult FIX-KO mice. Blood.

[14] Barzel A, Paulk NK, Shi Y, Huang Y, Chu K, Zhang F (2015). Promoterless gene targeting without nucleases ameliorates haemophilia B in mice. Nature.

[15] Sharma R, Anguela XM, Doyon Y, Wechsler T, DeKelver RC, Sproul S (2015). In vivo genome editing of the albumin locus as a platform for protein replacement therapy. Blood.

[16] Yin H, Song CQ, Dorkin JR, Zhu LJ, Li Y, Wu Q (2016). Therapeutic genome editing by combined viral and non-viral delivery of CRISPR system components in vivo. Nat Biotechnol.

[17] Nelson CE, Hakim CH, Ousterout DG, Thakore PI, Moreb EA, Rivera RMC (2016). In vivo genome editing improves muscle function in a mouse model of Duchenne muscular dystrophy. Science.

[18] Crystal RG (2014). Adenovirus: the first effective in vivo gene delivery vector. Hum Gene Ther.

[19] Li Q, Kay MA, Finegold M, Stratford-Perricaudet LD, Woo SL (1993). Assessment of recombinant adenoviral vectors for hepatic gene therapy. Hum Gene Ther.

[20] Le LP, Le HN, Nelson AR, Matthews DA, Yamamoto M, Curiel DT (2006). Core labeling of adenovirus with EGFP. Virology.

[21] Chartier C, Degryse E, Gantzer M, Dieterle A, Pavirani A, Mehtali M (1996). Efficient generation of recombinant adenovirus vectors by homologous recombination in Escherichia coli. J Virol.

[22] Maizel JV, David WO, Scharff MD (1968). The polypeptides of adenovirus: I. Evidence for multiple protein components in the virion and a comparison of types 2, 7A, and 12. Virology.

[23] Schmidt M, Schwarzwaelder K, Bartholomae C, Zaoui K, Ball C, Pilz I (2007). High-resolution insertion-site analysis by linear amplification-mediated PCR (LAM-PCR). Nat Methods.

[24] Kaliberov SA, Kaliberova LN, Lu ZH, Preuss MA, Barnes JA, Stockard CR (2013). Retargeting of gene expression using endothelium specific hexon modified adenoviral vector. Virology.

[25] Kieleczawa J (2006). Fundamentals of sequencing of difficult templates—an overview. J Biomol Technol.

[26] Nelson JE, Kay MA (1997). Persistence of recombinant adenovirus in vivo is not dependent on vector DNA replication. J Virol.

[27] Ehrhardt A, Xu H, Kay MA (2003). Episomal persistence of recombinant adenoviral vector genomes during the cell cycle in vivo. J Virol.

[28] Kay MA, Li Q, Liu TJ, Leland F, Toman C, Finegold M (1992). Hepatic gene therapy: persistent expression of human α1-antitrypsin in mice after direct gene delivery in vivo. Hum Gene Ther.

[29] Charlesworth CT, Deshpande PS, Dever DP, Dejene B, Gomez-Ospina N, Mantri S et al. Identification of pre-existing adaptive immunity to Cas9 proteins in humans. *bioRxiv* 2018; 243345.

[30] Chew WL, Tabebordbar M, Cheng JK, Mali P, Wu EY, Ng AH (2016). A multifunctional AAV-CRISPR-Cas9 and its host response. Nat Methods.

[31] Stephen SL, Montini E, Sivanandam VG, Al-Dhalimy M, Kestler HA, Finegold M (2010). Chromosomal integration of adenoviral vector DNA in vivo. J Virol.

[32] Dronadula N, Wacker BK, Van Der Kwast R, Zhang J, Dichek DA (2017). Stable in vivo transgene expression in endothelial cells with helper-dependent adenovirus: roles of promoter and interleukin-10. Hum Gene Ther.

[33] Iizuka S, Sakurai F, Tachibana M, Ohashi K, Mizuguchi H (2017). Neonatal gene therapy for hemophilia B by a novel adenovirus vector showing reduced leaky expression of viral genes. Mol Ther Methods Clin Dev.

[34] Kreppel F, Kochanek S (2004). Long-term transgene expression in proliferating cells mediated by episomally maintained high-capacity adenovirus vectors. J Virol.

[35] Yao X, Wang X, Hu X, Liu Z, Liu J, Zhou H (2017). Homology-mediated end joining-based targeted integration using CRISPR/Cas9. Cell Res.

[36] Nakade S, Tsubota T, Sakane Y, Kume S, Sakamoto N, Obara M (2014). Microhomology-mediated end-joining-dependent integration of donor DNA in cells and animals using TALENs and CRISPR/Cas9. Nat Commun.

[37] Malato Y, Naqvi S, Schürmann N, Ng R, Wang B, Zape J (2011). Fate tracing of mature hepatocytes in mouse liver homeostasis and regeneration. J Clin Invest.

[38] Tolmachov OE, Subkhankulova T, Tolmachova T. Silencing of transgene expression: a gene therapy perspective. In: Francisco MM (ed). Gene therapy—tools and potential applications. InTech; 2013.

[39] Hagedorn C, Antoniou M, Lipps HJ (2013). Genomic *cis*-acting sequences improve expression and establishment of a nonviral vector. Mol Ther Nucleic Acids.

[40] Irion S, Luche H, Gadue P, Fehling HJ, Kennedy M, Keller G (2007). Identification and targeting of the ROSA26 locus in human embryonic stem cells. Nat Biotechnol.

[41] Steinwaerder DS, Lieber A (2003). Insulation from viral transcriptional regulatory elements improves inducible transgene expression from adenovirus vectors in vitro and in vivo. Gene Therapy.

[42] Riu E, Chen ZY, Xu H, He CY, Kay MA (2007). Histone modifications are associated with the persistence or silencing of vector-mediated transgene expression in vivo. Mol Ther.

[43] Chen WY, Townes TM (2005). Molecular mechanism for silencing virally transduced genes involves histone deacetylation and chromatin condensation. Proc Natl Acad Sci USA.

[44] Chu VT, Weber T, Wefers B, Wurst W, Sander S, Rajewsky K (2015). Increasing the efficiency of homology-directed repair for CRISPR-Cas9-induced precise gene editing in mammalian cells. Nat Biotechnol.

[45] Maruyama T, Dougan SK, Truttmann M, Bilate AM, Ingram JR, Ploegh HL (2015). Inhibition of non-homologous end joining increases the efficiency of CRISPR/Cas9-mediated precise genome editing. Nat Biotechnol.

[46] Gwiazda KS, Grier AE, Sahni J, Burleigh SM, Martin U, Yang JG (2016). High efficiency CRISPR/Cas9-mediated gene editing in primary human T-cells using mutant adenoviral E4orf6/E1b55k “helper” proteins. Mol Ther.

[47] Baker O, Tsurkan S, Fu J, Klink B, Rump A, Obst M (2017). The contribution of homology arms to nuclease-assisted genome engineering. Nucleic Acids Res.

[48] Miyaoka Y, Berman JR, Cooper SB, Mayerl SJ, Chan AH, Zhang B (2016). Systematic quantification of HDR and NHEJ reveals effects of locus, nuclease, and cell type on genome-editing. Sci Rep.

[49] Kasparek P, Krausova M, Haneckova R, Kriz V, Zbodakova O, Korinek V (2014). Efficient gene targeting of the Rosa26 locus in mouse zygotes using TALE nucleases. FEBS Lett.

[50] Shin HY, Wang C, Lee HK, Yoo KH, Zeng X, Kuhns T (2017). CRISPR/Cas9 targeting events cause complex deletions and insertions at 17 sites in the mouse genome. Nat Commun.

[51] Schaefer KA, Wu WH, Colgan DF, Tsang SH, Bassuk AG, Mahajan VB (2017). Unexpected mutations after CRISPR-Cas9 editing in vivo. Nat Methods.

[52] Muruve DA, Barnes MJ, Stillman IE, Libermann TA (1999). Adenoviral gene therapy leads to rapid induction of multiple chemokines and acute neutrophil-dependent hepatic injury in vivo. Hum Gene Ther.

[53] Chandler RJ, Sands MS, Venditti CP (2017). Recombinant adeno-associated viral integration and genotoxicity: insights from animal models. Hum Gene Ther.

[54] Nault JC, Datta S, Imbeaud S, Franconi A, Mallet M, Couchy G (2015). Recurrent AAV2-related insertional mutagenesis in human hepatocellular carcinomas. Nat Genet.

[55] Deyle DR, Russell DW (2009). Adeno-associated virus vector integration. Curr Opin Mol Ther.

[56] Rutledge EA, Russell DW (1997). Adeno-associated virus vector integration junctions. J Virol.

[57] Donsante A, Miller DG, Li Y, Vogler C, Brunt EM, Russell DW (2007). AAV vector integration sites in mouse hepatocellular carcinoma. Science.

[58] Logan GJ, Dane AP, Hallwirth CV, Smyth CM, Wilkie EE, Amaya AK (2017). Identification of liver-specific enhancer-promoter activity in the 3’ untranslated region of the wild-type AAV2 genome. Nat Genet.

[59] Atasheva S, Shayakhmetov DM (2016). Adenovirus sensing by the immune system. Curr Opin Virol.

[60] Wang D, Mou H, Li S, Li Y, Hough S, Tran K (2015). Adenovirus-mediated somatic genome editing of Pten by CRISPR/Cas9 in mouse liver in spite of Cas9-specific immune responses. Hum Gene Ther.

[61] Kotini AG, Sadelain M, Papapetrou EP (2016). LiPS-A3S, a human genomic site for robust expression of inserted transgenes. Mol Ther Nucleic Acids.

[62] Sadelain M, Papapetrou EP, Bushman FD (2012). Safe harbours for the integration of new DNA in the human genome. Nat Rev Cancer.

[63] Yang Y, Wang L, Bell P, McMenamin D, He Z, White J (2016). A dual AAV system enables the Cas9-mediated correction of a metabolic liver disease in newborn mice. Nat Biotechnol.

[64] Crystal RG (1990). Alpha 1-antitrypsin deficiency, emphysema, and liver disease. Genetic basis and strategies for therapy. J Clin Inst.

[65] Holkers M, Maggio I, Henriques SF, Janssen JM, Cathomen T, Gonçalves MA (2014). Adenoviral vector DNA for accurate genome editing with engineered nucleases. Nat Methods.

[66] Richter M, Saydaminova K, Yumul R, Krishnan R, Liu J, Nagy EE (2016). In vivo transduction of primitive mobilized hematopoietic stem cells after intravenous injection of integrating adenovirus vectors. Blood.

[67] Verweij CL (1988). Biosynthesis of human von Willebrand factor. Haemostasis.

[68] Fahs SA, Hille MT, Shi Q, Weiler H, Montgomery RR (2014). A conditional knockout mouse model reveals endothelial cells as the principal and possibly exclusive source of plasma factor VIII. Blood.

[69] Chen CM, Krohn J, Bhattacharya S, Davies B (2011). A comparison of exogenous promoter activity at the ROSA26 locus using a PhiC31 integrase mediated cassette exchange approach in mouse ES cells. PLoS ONE.

[70] Monahan PE, Sun GJ, Gui T, Hu G, Hannah WB, Wichlan DG (2014). Employing a gain-of-function factor IX variant R338L to advance the efficacy and safety of hemophilia B human gene therapy: preclinical evaluation supporting an ongoing adeno-associated virus clinical trial. Hum Gene Ther.

[71] Nguyen GN, George LA, Siner JI, Davidson RJ, Zander CB, Zheng XL (2017). Novel factor VIII variants with a modified furin cleavage site improve the efficacy of gene therapy for hemophilia A. J Thromb Haemost.

