# Supplementary material for: Targeted in vivo knock-in of human alpha-1-antitrypsin cDNA using adenoviral delivery of CRISPR/Cas9
Source: Gene Ther. 2018 Mar 27;25(2):139–56. doi: 10.1038/s41434-018-0003-1 (PMC5919923; doi:10.1038/s41434-018-0003-1)
Supplement: Supplementary file 1 — Supplemental Table 1(PDF 280 kb) [file 41434_2018_3_MOESM1_ESM.pdf]

Primers for Analysis of ROSA26 Gene Editing			
**ID**	**Primer Binding Target**	**Sequence**	**PCR Amplicon**
1	EF1α promoter (5' end, Reverse Orientation)	TTCTCTAGGCACCGGTTCAATTGC	5' Junction
2	*EGFP* (3'end, Forward Orientation)	GTCCTGCTGGAGTTCGTGACC	3' Junction
3	*ROSA26* locus (5' end, Forward orientation, outside of homology arms)	CTGATTGGCTTCTTTTCCTCCCGCC	5' Junction
4	Ad5 genomic location 18452-18472	CGTACGAGGAGGCACTAAAGC	Internal Ad5 Hexon gene
5	Ad5 genomic location in Hexon gene	CATCATCGAAGGGGTAGCCAT	Internal Ad5 Hexon gene
6	*ROSA26* locus (3' end, Reverse orientation, past homology arms)	GAGTCAAGCCAGTCCAAGAGAAAGC	3' Junction
7	*hAAT* (3' end, Forward orientation)	CTCCAAGGCCGTGAAGG	3' Junction
8	*ROSA26* locus (3' end, Reverse orientation, past homology arms)	AAGCTCACAAGACCTTAGGTCAGGAAAGAC	3' Junction
9	*EGFP* (Forward orientation)	GAAGCAGCACGACTTCTTCAAG	*EGFP* qPCR
10	*EGFP* (Reverse orientation)	AAGTCGATGCCCTTCAGCTC	*EGFP* qPCR
11	*EGFP* (Taqman Probe)	[6FAM]CAAGGACGACGGCAACTACAAGACCCGC[BHQ1]	*EGFP* qPCR
12	*Hexon* (Forward orientation)	TACGCACGACGTGACCACA	*Hexon* qPCR
13	*Hexon* (Reverse orientation)	ATCCTCACGGTCCACAGGG	*Hexon* qPCR
14	*Hexon* (Taqman Probe)	[6FAM]ACCGGTCCCAGCGTTTGACGC[BHQ1]	*Hexon* qPCR
15	*mActin* (Forward orientation)	AGCTGGAGGACTTCCGAGACT	*mActin* qPCR
16	*mActin* (Reverse orientation)	TGGCACTTCTCCTGCACCTT	*mActin* qPCR
17	*mActin* (Taqman Probe)	[HEX]TAGACGCCTGCACAAGCCGCC[BHQ1]	*mActin* qPCR
18	*ROSA26* (3' end, reverse orientation)	[Btn]GAGTCAAGCCAGTCCAAGAGAAAGC	LAM-PCR linear amplification
19	Plus Orientation Linker (5' to 3'), anneals	5'-3' CTGAAGGGCTCAGGTTACACA GGCACGCTCGTAGGAGGTGTTCCAGTTCACCACG	LAM-PCR, Linker

	negative orientation linker		
20	Negative Orientation Linker (3’ to 5’), anneals positive orientation linker	3’-5’ [3d_C]CGAGCCTCCACAAGGTCAAGTGGTGCGTAC	LAM-PCR, Linker
21	LAM-PCR Linker, Forward orientation	CTGAAGGCTCAGGTTACACAGGCAC	LAM-PCR, 1st nested PCR
22	*ROSA26,* 3’ end reverse orientation (outside of right homology arm)	AAGCTCACAAGACCTTAGGTCAGGAAAGAC	LAM-PCR, 1st nested PCR
23	LAM-PCR Linker, Forward orientation	GCTCGTAGGAGGTGTTCCAGTTCACC	LAM-PCR, 2nd nested PCR
24	*ROSA26,* 3’ end reverse orientation (inside right homology arm)	GAAGGAGCGGGAGAAATGGATATGAAG	LAM-PCR, 2nd nested PCR
25	*ROSA26,* 5’ end, forward orientation (inside left homology arm)	GGAGGGTCAGCGAAAGTAGCTC	LAM-PCR, Wild-type qPCR
26	*ROSA26,* 5’ end, reverse orientation (inside left homology arm)	ATGGACTCAACTTGCACGAACAC	LAM-PCR, Wild-type qPCR
27	*ROSA26,* 5’ end (inside left homology arm)	[6FAM]CTTCCTCTGGGGGAGTCGTTTTACCCGCC[BHQ1]	LAM-PCR, Wild-type qPCR
