# Supplementary material for: Targeted in vivo knock-in of human alpha-1-antitrypsin cDNA using adenoviral delivery of CRISPR/Cas9
Source: Gene Ther. 2018 Mar 27;25(2):139–56. doi: 10.1038/s41434-018-0003-1 (PMC5919923; doi:10.1038/s41434-018-0003-1)
Supplement: Supplementary file 2 — Supplemental Table 2(PDF 51 kb) [file 41434_2018_3_MOESM2_ESM.pdf]

# Supplemental Table 2A

| I.D. | Treatment | Dose | Total Reads | #1-Indel (W/T) | #1-Reads(%) | #2-Indel | #2-Reads(%) | #3-Indel | #3-Reads(%) | #4-Indel | #4-Reads(%) | #5-Indel | #5-Reads(%) | #6-Indel | #6-Reads(%) | #7-Indel | #7-Reads(%) | #8-Indel | #8-Reads(%) |
|---|---|---|---|---|---|---|---|---|---|---|---|---|---|---|---|---|---|---|---|
| 1 | PBS | 0 VP/Cell | 238 | 0 | 237 (99.6%) | -1 | 1 (0.4%) | NA | | NA | | NA | | NA | | NA | | NA | |
| 2 | PBS | 0 VP/Cell | 388 | 0 | 387 (99.7%) | -3 | 1 (0.3%) | NA | | NA | | NA | | NA | | NA | | NA | |
| 3 | Ad5-Cas9-gRNA | 1 x $10^3$ VP/cell | 318 | 0 | 309 (97.2%) | -1 | 5 (1.6%) | -15 | 2 (0.6%) | -118 | 1 (0.3%) | -68 | 1 (0.3%) | NA | | NA | | NA | |
| 4 | Ad5-Cas9-gRNA | 1 x $10^3$ VP/cell | 453 | 0 | 438 (96.7%) | -1 | 6 (1.3%) | -3 | 2 (0.4%) | -19 | 1 (0.2%) | -17 | 1 (0.2%) | -15 | 1 (0.2%) | -2 | 1 (0.2%) | -41 | 1 (0.2%) |
| 5 | Ad5-Cas9-gRNA | 2 x $10^3$ VP/cell | 362 | 0 | 343 (94.8%) | -15 | 3 (0.8%) | -125 | 2 (0.6%) | -14 | 2 (0.6%) | -3 | 2 (0.6%) | -1 | 2 (0.6%) | -28 | 1 (0.3%) | -91 | 1 (0.3%) |
| 6 | Ad5-Cas9-gRNA | 2 x $10^3$ VP/cell | 339 | 0 | 322 (95.0%) | -15 | 4 (1.2%) | -1 | 3 (0.9%) | -2 | 2 (0.6%) | -3 | 2 (0.6%) | -112 | 1 (0.3%) | -54 | 1 (0.3%) | -16 | 1 (0.3%) |
| 7 | Ad5-Cas9-gRNA | 4 x $10^3$ VP/cell | 439 | 0 | 417 (95.0%) | -15 | 5 (1.1%) | -1 | 4 (0.9%) | -74 | 3 (0.7%) | -2 | 3 (0.7%) | -12 | 2 (0.5%) | -95 | 1 (0.2%) | -27 | 1 (0.2%) |
| 8 | Ad5-Cas9-gRNA | 4 x $10^3$ VP/cell | 537 | 0 | 480 (89.4%) | -15 | 16 (3.0%) | -1 | 8 (1.5%) | -2 | 4 (0.7%) | -19 | 3 (0.6%) | -108 | 2 (0.4%) | -80 | 2 (0.4%) | -43 | 2 (0.4%) |
| 9 | Ad5-Cas9-gRNA | 6 x $10^3$ VP/cell | 361 | 0 | 301 (83.4%) | -15 | 16 (4.4%) | -1 | 11 (3.0%) | -2 | 5 (1.4%) | -3 | 4 (1.1%) | -80 | 2 (0.6%) | -79 | 2 (0.6%) | -19 | 2 (0.6%) |
| 10 | Ad5-Cas9-gRNA | 6 x $10^3$ VP/cell | 281 | 0 | 226 (80.4%) | -15 | 12 (4.3%) | -1 | 9 (3.2%) | -2 | 4 (1.4%) | -3 | 3 (1.1%) | 1 | 2 (0.7%) | -79 | 2 (0.7%) | -43 | 2 (0.7%) |
| 11 | Ad5-Cas9-gRNA | 8 x $10^3$ VP/cell | 501 | 0 | 390 (77.8%) | -1 | 24 (4.8%) | -15 | 16 (3.2%) | -2 | 10 (2.0%) | -3 | 8 (1.6%) | 1 | 7 (1.4%) | -26 | 4 (0.8%) | -95 | 3 (0.6%) |
| 12 | Ad5-Cas9-gRNA | 8 x $10^3$ VP/cell | 388 | 0 | 310 (79.9%) | -15 | 13 (3.4%) | -1 | 12 (3.1%) | 1 | 4 (1.0%) | -116 | 3 (0.8%) | -114 | 3 (0.8%) | -16 | 3 (0.8%) | -2 | 3 (0.8%) |
| 13 | Ad5-Cas9-gRNA | 1 x $10^4$ VP/cell | 350 | 0 | 255 (72.9%) | -15 | 17 (4.9%) | -2 | 14 (4.0%) | -1 | 12 (3.4%) | 1 | 6 (1.7%) | -3 | 5 (1.4%) | -19 | 4 (1.1%) | -66 | 3 (0.9%) |
| 14 | Ad5-Cas9-gRNA | 1 x $10^4$ VP/cell | 211 | 0 | 156 (73.9%) | -15 | 9 (4.3%) | -1 | 9 (4.3%) | -3 | 6 (2.8%) | 1 | 3 (1.4%) | -2 | 3 (1.4%) | -80 | 2 (0.9%) | -23 | 2 (0.9%) |
| 15 | Ad5-Cas9-gRNA | 2 x $10^4$ VP/cell | 346 | 0 | 184 (53.2%) | -1 | 35 (10.1%) | -15 | 22 (6.4%) | -2 | 15 (4.3%) | -3 | 9 (2.6%) | -19 | 5 (1.4%) | -79 | 4 (1.2%) | 1 | 3 (0.9%) |
| 16 | Ad5-Cas9-gRNA | 2 x $10^4$ VP/cell | 462 | 0 | 271 (58.7%) | -1 | 35 (7.6%) | -15 | 32 (6.9%) | -2 | 24 (5.2%) | -3 | 17 (3.7%) | -35 | 5 (1.1%) | -19 | 5 (1.1%) | -22 | 4 (0.9%) |

# Supplemental Table 2B

| I.D. | Treatment | Dose | Total Reads | #1-Indel (W/T) | #1-Reads(%) | #2-Indel | #2-Reads(%) | #3-Indel | #3-Reads(%) | #4-Indel | #4-Reads(%) | #5-Indel | #5-Reads(%) | #6-Indel | #6-Reads(%) | #7-Indel | #7-Reads(%) | #8-Indel | #8-Reads(%) |
|---|---|---|---|---|---|---|---|---|---|---|---|---|---|---|---|---|---|---|---|
| 2 | PBS | 0 VP | 578 | 0 | 567 (98.1%) | -1 | 10 (1.7%) | -2 | 1 (0.2%) | NA | | NA | | NA | | NA | | NA | |
| 3 | Ad5-EF1α-eGFP + Ad5-gRNA | 5 x $10^{10}$ VP (each vector) | 3629 | 0 | 3556 (98.0%) | -1 | 63 (1.7%) | -2 | 9 (0.2%) | 1 | 1 (0.0%) | NA | | NA | | NA | | NA | |
| 4 | Ad5-EF1α-eGFP + Ad5-gRNA | 5 x $10^{10}$ VP (each vector) | 3486 | 0 | 3422 (98.2%) | -1 | 52 (1.5%) | 46 | 6 (0.2%) | -2 | 3 (0.1%) | 1 | 2 (0.1%) | 2 | 1 (0.0%) | NA | | NA | |
| 5 | Ad5-EF1α-eGFP + Ad5-gRNA | 5 x $10^{10}$ VP (each vector) | 4269 | 0 | 4169 (97.7%) | -1 | 89 (2.1%) | -2 | 7 (0.2%) | 1 | 4 (0.1%) | NA | | NA | | NA | | NA | |
| 6 | Ad5-EF1α-eGFP + Ad5-gRNA | 5 x $10^{10}$ VP (each vector) | 539 | 0 | 526 (97.6%) | -1 | 12 (2.2%) | -2 | 1 (0.2%) | NA | | NA | | NA | | NA | | NA | |
| 7 | Ad5-EF1α-eGFP + Ad5-Cas9-gRNA | 5 x $10^{10}$ VP (each vector) | 3649 | 0 | 3473 (95.2%) | -1 | 88 (2.4%) | 46 | 21 (0.6%) | -2 | 20 (0.5%) | 1 | 12 (0.3%) | -15 | 3 (0.1%) | -3 | 3 (0.1%) | 2 | 2 (0.1%) |
| 9 | Ad5-EF1α-eGFP + Ad5-Cas9-gRNA | 5 x $10^{10}$ VP (each vector) | 3460 | 0 | 3273 (94.6%) | -1 | 88 (2.5%) | 1 | 17 (0.5%) | -2 | 16 (0.5%) | -3 | 8 (0.2%) | -19 | 4 (0.1%) | -15 | 4 (0.1%) | -120 | 3 (0.1%) |
| 10 | Ad5-EF1α-eGFP + Ad5-Cas9-gRNA | 5 x $10^{10}$ VP (each vector) | 3249 | 0 | 3091 (95.1%) | -1 | 75 (2.3%) | -2 | 20 (0.6%) | -3 | 15 (0.5%) | 1 | 9 (0.3%) | -15 | 5 (0.2%) | -34 | 3 (0.1%) | -11 | 2 (0.1%) |
| 11 | Ad5-EF1α-eGFP + Ad5-Cas9-gRNA | 5 x $10^{10}$ VP (each vector) | 5431 | 0 | 5212 (96.0%) | -1 | 122 (2.2%) | -2 | 27 (0.5%) | 1 | 14 (0.3%) | -3 | 6 (0.1%) | -15 | 4 (0.1%) | -103 | 3 (0.1%) | -66 | 3 (0.1%) |
| 12 | Ad5-EF1α-eGFP + Ad5-Cas9-gRNA | 5 x $10^{10}$ VP (each vector) | 3649 | 0 | 3471 (95.1%) | -1 | 86 (2.4%) | -2 | 30 (0.8%) | -3 | 9 (0.2%) | 1 | 8 (0.2%) | -15 | 6 (0.2%) | -4 | 5 (0.1%) | -5 | 3 (0.1%) |
| 13 | Ad5-EF1α-eGFP + Ad5-Cas9-gRNA | 5 x $10^{10}$ VP (each vector) | 4509 | 0 | 4095 (90.8%) | -1 | 138 (3.1%) | -2 | 47 (1.0%) | 1 | 35 (0.8%) | -15 | 23 (0.5%) | -3 | 23 (0.5%) | -4 | 10 (0.2%) | 2 | 9 (0.2%) |
| 14 | Ad5-EF1α-eGFP + Ad5-Cas9-gRNA | 5 x $10^{10}$ VP (each vector) | 1026 | 0 | 919 (89.6%) | -1 | 43 (4.2%) | 1 | 9 (0.9%) | -15 | 9 (0.9%) | -2 | 9 (0.9%) | -3 | 7 (0.7%) | -18 | 4 (0.4%) | -27 | 3 (0.3%) |
| 15 | Ad5-EF1α-eGFP + Ad5-Cas9-gRNA | 5 x $10^{10}$ VP (each vector) | 3515 | 0 | 3349 (95.3%) | -1 | 84 (2.4%) | -2 | 16 (0.5%) | 1 | 10 (0.3%) | -15 | 7 (0.2%) | -4 | 4 (0.1%) | -3 | 4 (0.1%) | 46 | 3 (0.1%) |
