# Supplementary material for: Targeted in vivo knock-in of human alpha-1-antitrypsin cDNA using adenoviral delivery of CRISPR/Cas9
Source: Gene Ther. 2018 Mar 27;25(2):139–56. doi: 10.1038/s41434-018-0003-1 (PMC5919923; doi:10.1038/s41434-018-0003-1)
Supplement: Supplementary file 3 — Supplemental Figure Legends(DOCX 15 kb) [file 41434_2018_3_MOESM3_ESM.docx]

**Supplemental Figure Legends

Supplemental Figure 1. Flow cytometry and standard curves used for quantitative studies.** (A) Side and forward size scattering gating was performed on uninfected PBS cells, as well as determination of ‘GFP +’ positivity. Left image is PBS treated cells; middle image is cells transduced with 4x10^3^ VP/cell Ad5-Cas9 + 4x10^3^ Ad5-EF1α-EGFP; right image is cells transduced with 4x10^3^ VP/cell Ad5-Cas9-gRNA + 4x10^3^ Ad5-EF1α-EGFP, all from 36 dpi after a long-term culture of four passages. (B) Initial GFP+ percent of cells used for sorting transduced BNL-1NG for use in the long-term culture experiment. (C) Representative amplification curves of EGFP integrated (left) and Wild-type (right) *ROSA26* alleles in qPCR following LAM-PCR. (D) Linearity of representative qPCR standard curves used for quantitation of EGFP (top) and Wild-Type (bottom) *ROSA26* alleles following LAM-qPCR.

**Supplemental Figure 2. hAAT ELISA specifications to determine serum concentrations.** (A) The dynamic range of hAAT ELISA assay was determined by serial ten-fold dilution of recombinant hAAT run in triplicate, showing a linear range between approximately 0.4 to 1.8 OD at 450nm. (B) Graph showing the linearity of standard curves from all plates used to quantitate hAAT plasma levels for the entire time course of Figure 3. (C) Cross reactivity of polyclonal goat anti-hAAT capture antibody was determined to be minimal against mouse plasma (PBS-injected) and BSA diluent.

**Supplemental Figure 3. Sanger sequencing of the 5’ Junction PCR amplicons validates on-target insertion.** (A) A junction capture PCR for amplification of the 5’ *EGFP*-integrated *ROSA26* locus was performed with a primer binding within the EF1α promoter in reverse orientation and a primer within the *ROSA26* genomic locus past the left homology arm (see Figure 2B). The PCR amplicon was gel extracted, purified, and submitted to Sanger sequencing in two separate reactions (one for each primer). Sequencing of the amplicon with the primer annealing in the EF1α promoter shows amplification of the promoter, cloning sites from vector construction, and the left homology arm from the junction amplicon. (B) Sequencing of the amplicon using the primer annealing in the upstream 5’ genomic region shows amplification of the genomic region past the left homology arm and the left homology arm before the peaks drop off dramatically at an extremely GC-rich region, potentially caused by polymerase stalling at secondary structures.

**Supplemental Figure 4. Sanger sequencing of the 3’ Junction PCR amplicons validates on-target insertion.** A junction capture PCR for amplification of the 3’ *EGFP*-integrated *ROSA26* locus was performed with a primer annealing within the 3’ end of the *EGFP* gene in forward orientation and a primer within the *ROSA26* genomic locus past the right homology arm (see Figure 2B). The PCR amplicon was gel extracted, purified, and submitted to Sanger sequencing in two separate reactions (one for each primer). (A) Sequencing of the amplicon with the primer annealing in the *EGFP* gene shows amplification of *EGFP* cDNA, poly-(A) region, sequences from cloning plasmids used in donor vector construction, and most of the right homology. (B) Sequencing of the amplicon using the primer annealing in the downstream 3’ genomic region shows amplification of the genomic region past the right homology arm, the entire right homology arm, and the beginning of sequences from the plasmids used in donor vector construction.

**Supplemental Figure 5. In vitro studies of cellular proliferation and vector dilution to determine integrations role in gene maintenance.** (A) BNL-1NG cells were transduced with Ad5-EF1α-EGFP and Ad5-Cas9 or Ad5-EF1α-EGFP and Ad5-Cas9-gRNA. 5 dpi cells were sorted by GFP positivity to select transduced cells (Supplemental Figure 1A), and equal numbers of GFP+ cells were cultured for 36 days to dilute out episomes during cell division. Remaining GFP+ cell populations were then quantified via flow cytometry. Two vector doses (8,000 total VP/cell and 12,000 total VP/cell) at 1:1 ratios of (Ad5-EF1α-EGFP: Ad5-Cas9 or Ad5-EF1α-EGFP: Ad5-Cas9-gRNA) were tested. Data is representative of four independent experiments. Error bars are standard deviation (s.d.) of the mean. N.S. represents non-significant differences with a p-value >0.05. (B) Equal numbers of GFP+ cells from the 36 day culture were seeded for a three day recovery and expansion. Whole DNA was then extracted for qPCR quantification of the *EGFP* gene to enumerate integrated and non-integrated transgene copies and the *hexon* gene to enumerate remaining adenoviral genome copy numbers. Copy numbers were normalized to the *m-actin* gene. Lines shown are mean of all data points, error bars are the s.d. of the mean.

**Supplemental Figure 6. Injection of Ad5-CMV-hAAT non-integrative vector shows persistent gene expression of the hAAT serum protein, in the absence of any integrative components.** (A) Two mice were injected with 1 x 10^11^ viral particles of Ad5-CMV-hAAT, a vector expressing *hAAT* cDNA from the CMV promoter and lacking any CRISPR/Cas9 integration components, at the same time as mice in Figure 3A. Plasma was collected and analyzed via ELISA at the same time as samples in 3A.

**Supplemental Figure 7. Adenoviral vectors display high transduction witnessed by reporter gene expression *in vivo*.** (A) Mice were injected with PBS (left column), 5x10^10^ VP Ad5-EF1α-EGFP and 5x10^10^ VP Ad5-gRNA (middle column), or 5x10^10^ VP Ad5-EF1α-EGFP and 5x10^10^ VP Ad5-Cas9-gRNA (right column). At 7, 21, and 42 dpi one mouse per group was sacrificed, perfused with formalin, and livers frozen embedded for indirect IHC staining of EGFP expression.

**Supplemental Figure 8. Targeted deep sequencing of *ROSA26* locus shows indel formation in a time-dependent manner.**  Mice receiving 5 x10^10^ VP of Ad5-Cas9-gRNA were sacrificed at various time points from 3 dpi to 210 dpi, whole genomic DNA was extracted from the liver, and submitted to targeted Illumina deep sequencing of *ROSA26.* Each dot represents data from one mouse.

**Supplemental Figure 9. Gene editing occurs mostly in liver following Ad5-Cas9-gRNA injection.** (A) One mouse was injected with 1x10^11^ VP of Ad5-Cas9-gRNA, sacrificed 72 hours later, and whole genomic DNA was extracted from the liver, kidney, and spleen. Genomic DNA was submitted to targeted Illumina deep sequencing of the *ROSA26* locus demonstrating the majority of gene editing was occurring in the liver. Data was normalized to uninfected mouse liver genomic DNA.

**Supplemental Figure 10. Standard analysis of biomarkers reveals no overt consequences of genetic editing after thirty weeks after injection of viral vectors.** (A) Mice receiving PBS (negative control), hAAT donor and GFP expressing sham vector, or hAAT donor and CRISPR/Cas9-expressing vectors were weighed at the conclusion of the experiment. Weights between three groups did not vary significantly from PBS mock injected mice (N.S. denotes not significant p-value >0.05). Two groups were significantly less than PBS mock injected control mice (* denotes significant with a p-value <.05). Statistical testing was performed with paired and unpaired two tailed student T-tests (in agreement). The mice were not weighed prior to injection. (B) Liver organ weight was measured at thirty weeks (200 days+) and expressed as a percentage of total body weight. The mouse with the smaller percentage of liver weight to total body weight was from the mouse group containing the largest mice (C) Analysis of cellular blood markers for anemia and thalassemia between mouse groups. Large variation in PVC for PBS mice was attributed to mild hemolysis of sample from one mouse. (D) Analysis of white blood cell population differentials in peripheral blood smears between mice receiving PBS, non-integrative, or integrative adenoviral vectors at thirty weeks post injection. . Each dot represents data from one mouse for panels A through D.

**Supplemental Table 2. Targeted deep sequencing of *ROSA26* locus reveals various sizes of indel formation.** (A) Table representative of the types of indel alleles detected at the *ROSA26* locus following Illumina deep sequencing of genomic DNA samples from BNL-1NG cells treated with PBS (no virus) or Ad5-Cas9-gRNA at various doses. Each indel is either an insertion (e.g. 2) or deletion (e.g. -2). Each read signifies one indel type, with read #1 representing no indel/mutation or wild-type (W/T) *ROSA26* sequence read. Abundance of each indel allele is expressed as % of total reads per sample. (B) Table representative of indel formations detected via Illumina deep sequencing of gDNA from livers of mice receiving no virus, Ad5-EF1α-EGFP and Ad5-gRNA, or Ad5-EF1α-EGFP and Ad5-Cas9-gRNA. Mice were sacrificed at 7, 21, and 42 days post-infection and genomic DNA extracted for sequencing.
