# Supplementary figures and images for: Targeted in vivo knock-in of human alpha-1-antitrypsin cDNA using adenoviral delivery of CRISPR/Cas9
Source: Gene Ther. 2018 Mar 27;25(2):139–56. doi: 10.1038/s41434-018-0003-1 (PMC5919923; doi:10.1038/s41434-018-0003-1)

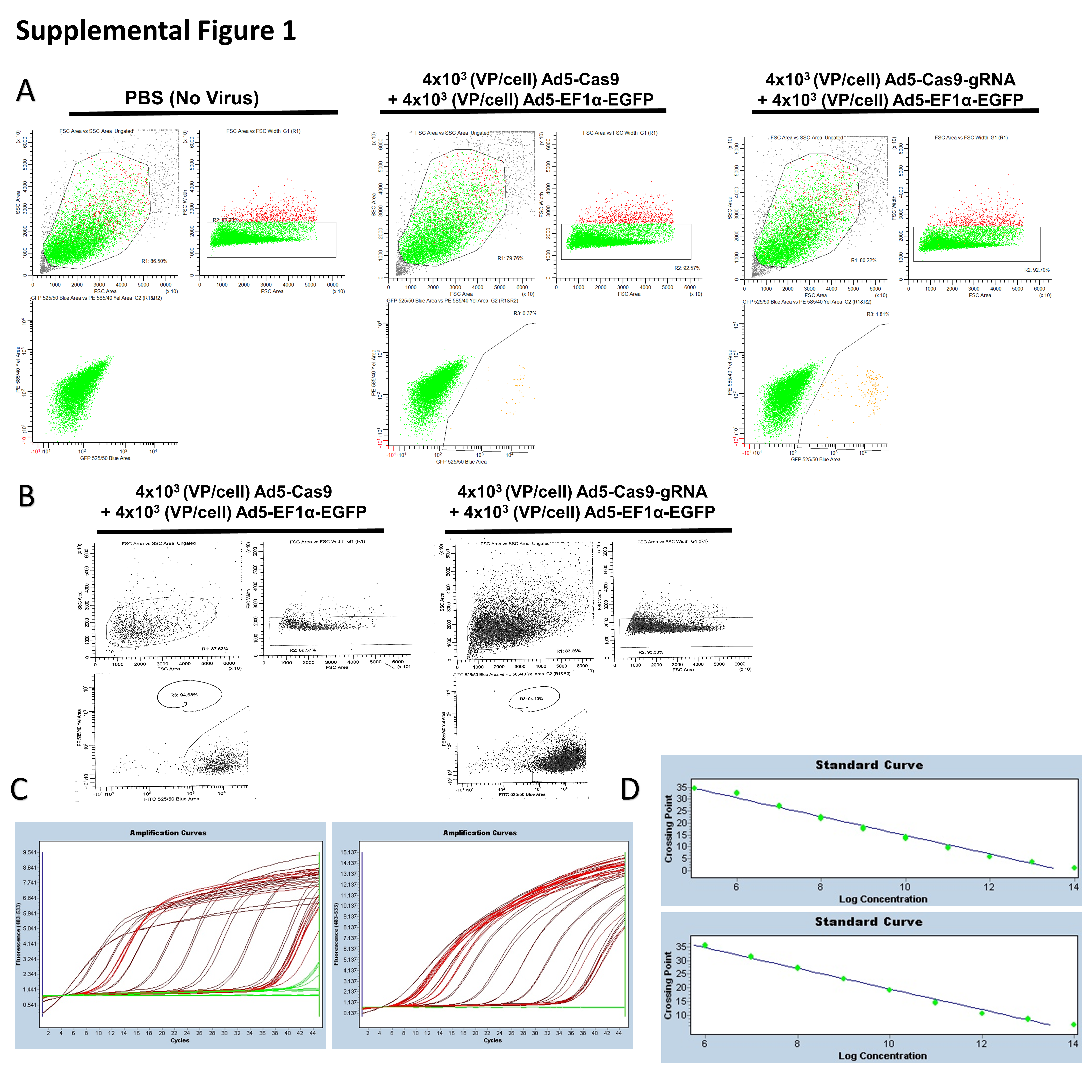

Supplement: Supplementary file 4 — Supplemental Figure 1(TIF 12508 kb) [file 41434_2018_3_MOESM4_ESM.tif]

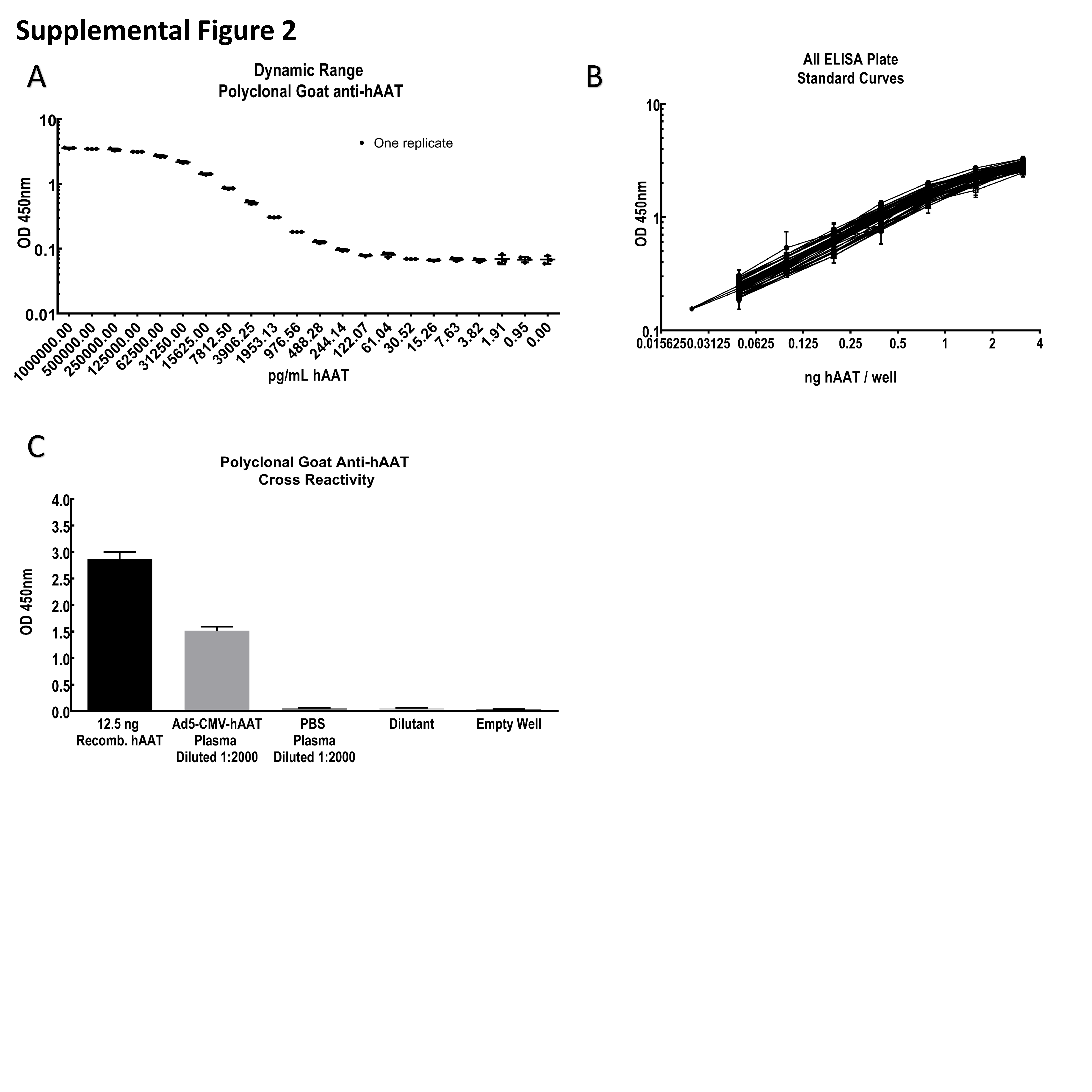

Supplement: Supplementary file 5 — Supplemental Figure 2(TIF 3245 kb) [file 41434_2018_3_MOESM5_ESM.tif]

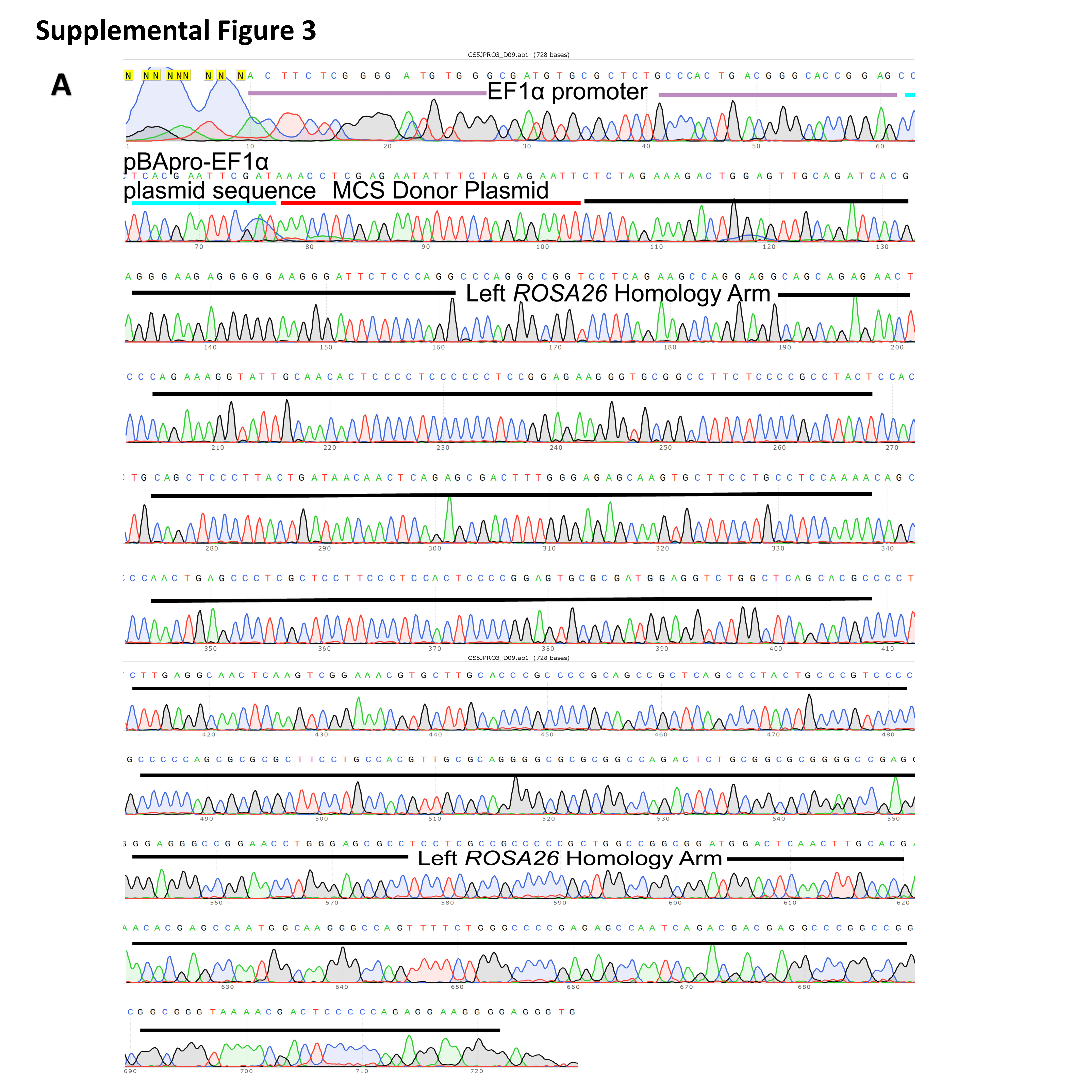

Supplement: Supplementary file 6 — Supplemental Figure 3 A(TIF 17052 kb) [file 41434_2018_3_MOESM6_ESM.tif]

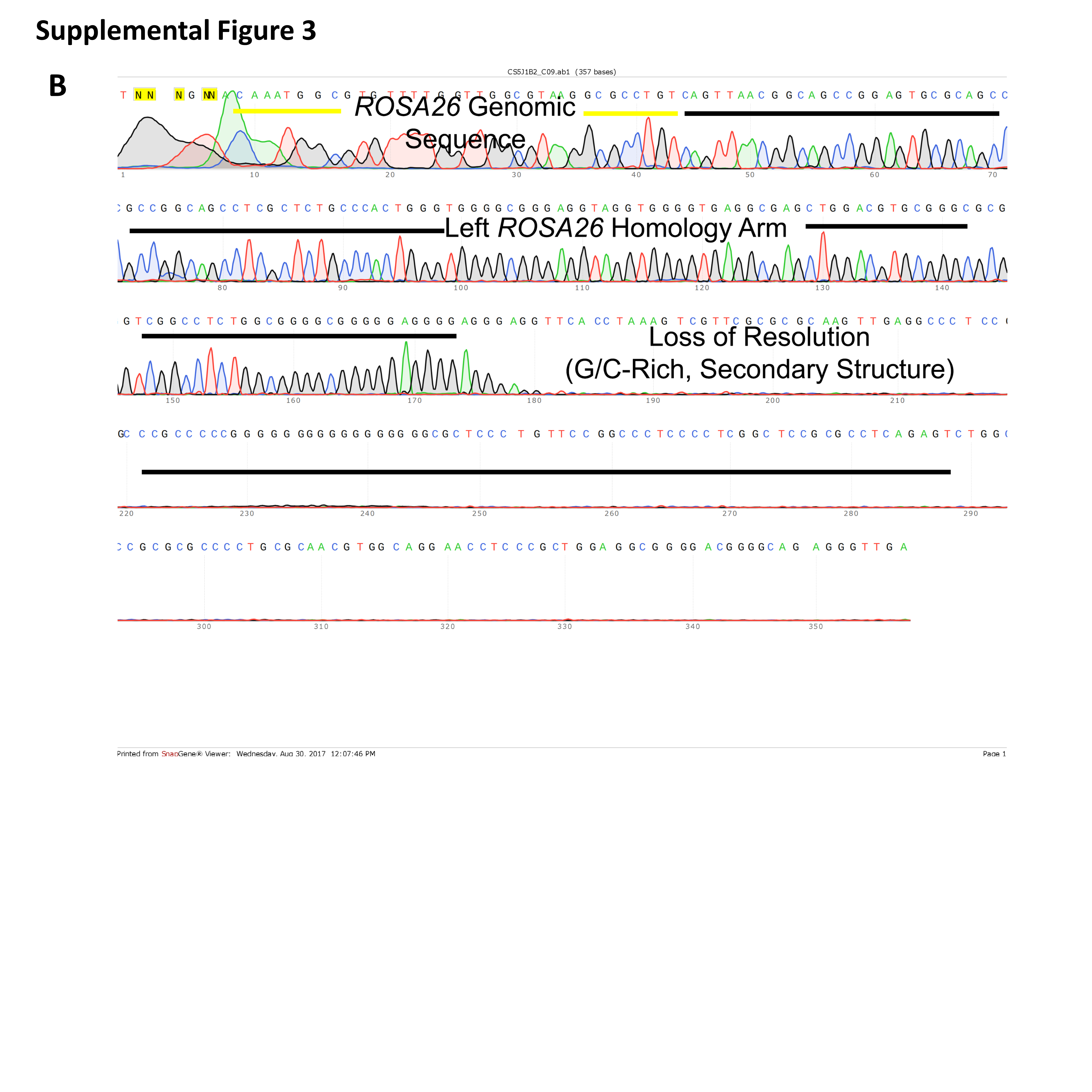

Supplement: Supplementary file 7 — Supplemental Figure 3 B(TIF 6613 kb) [file 41434_2018_3_MOESM7_ESM.tif]

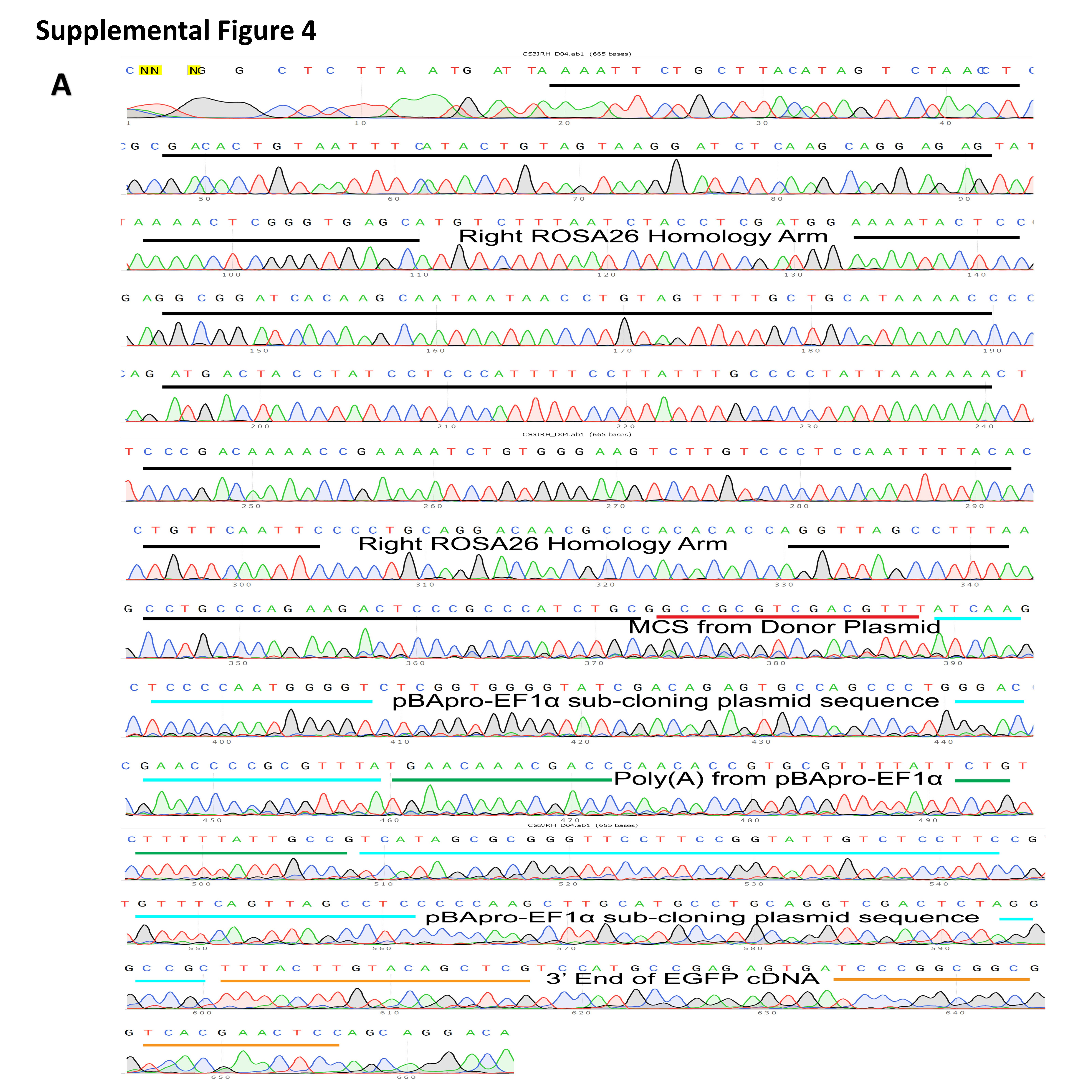

Supplement: Supplementary file 8 — Supplemental Figure 4 A(TIF 16140 kb) [file 41434_2018_3_MOESM8_ESM.tif]

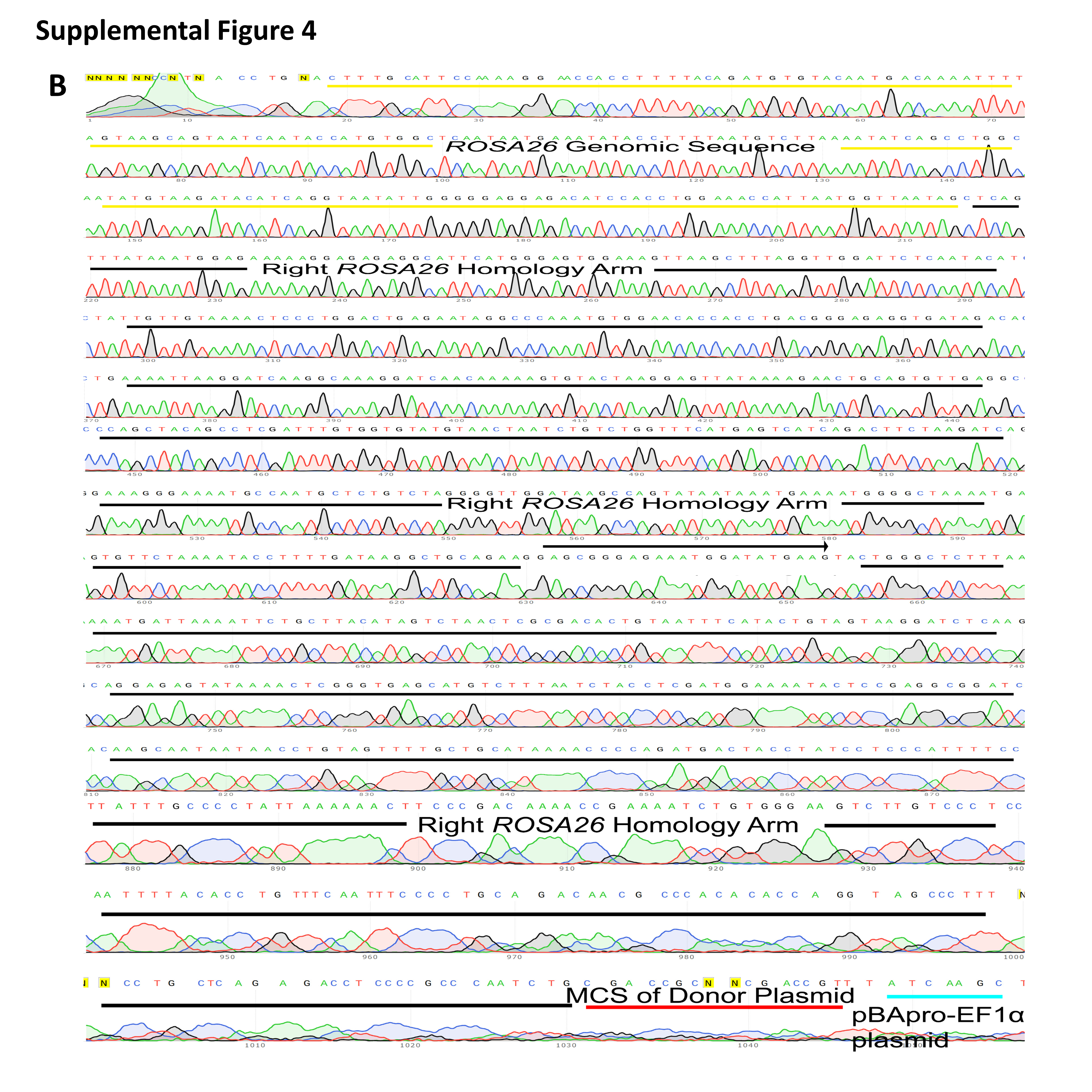

Supplement: Supplementary file 9 — Supplemental Figure 4 B(TIF 16274 kb) [file 41434_2018_3_MOESM9_ESM.tif]

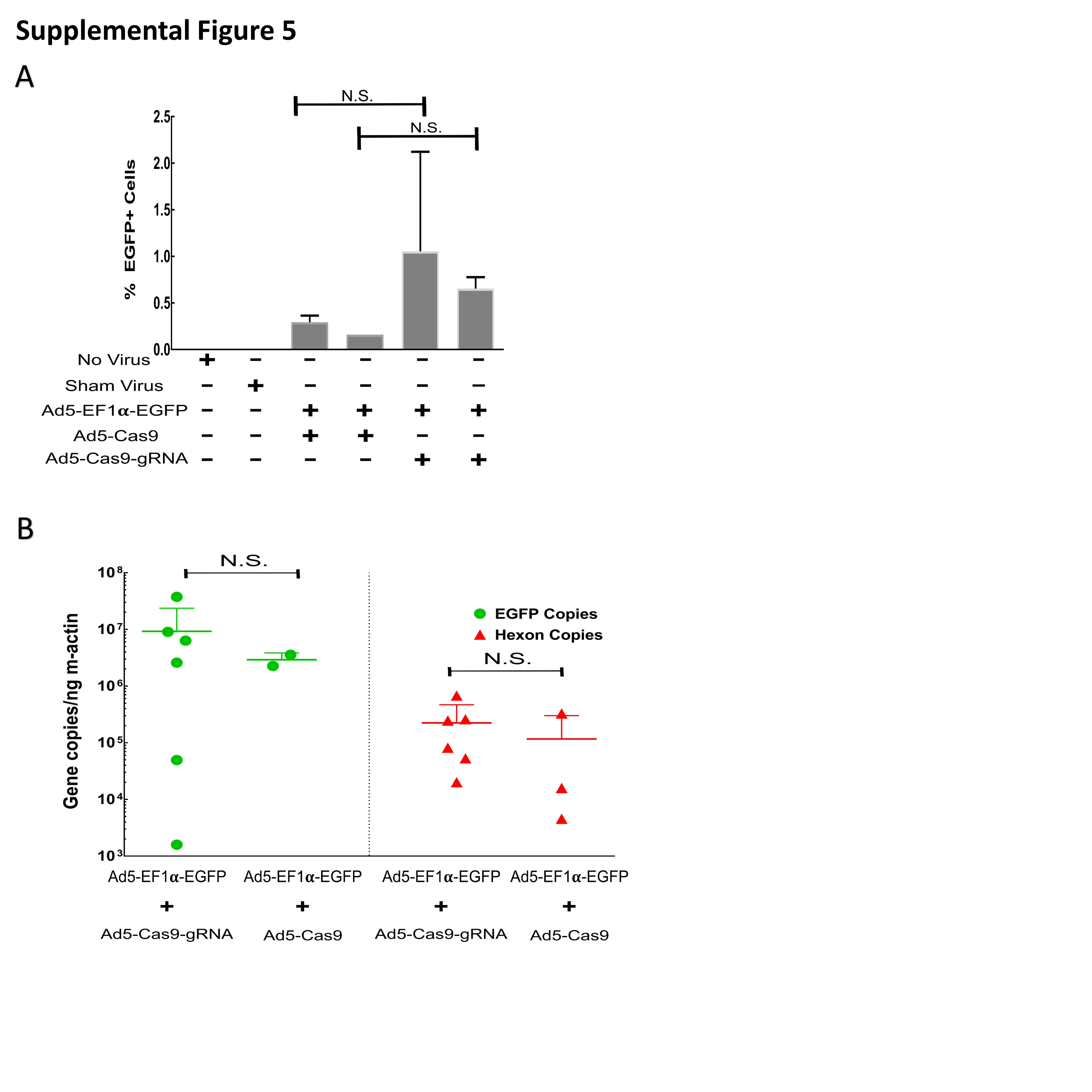

Supplement: Supplementary file 10 — Supplemental Figure 5(TIF 2768 kb) [file 41434_2018_3_MOESM10_ESM.tif]

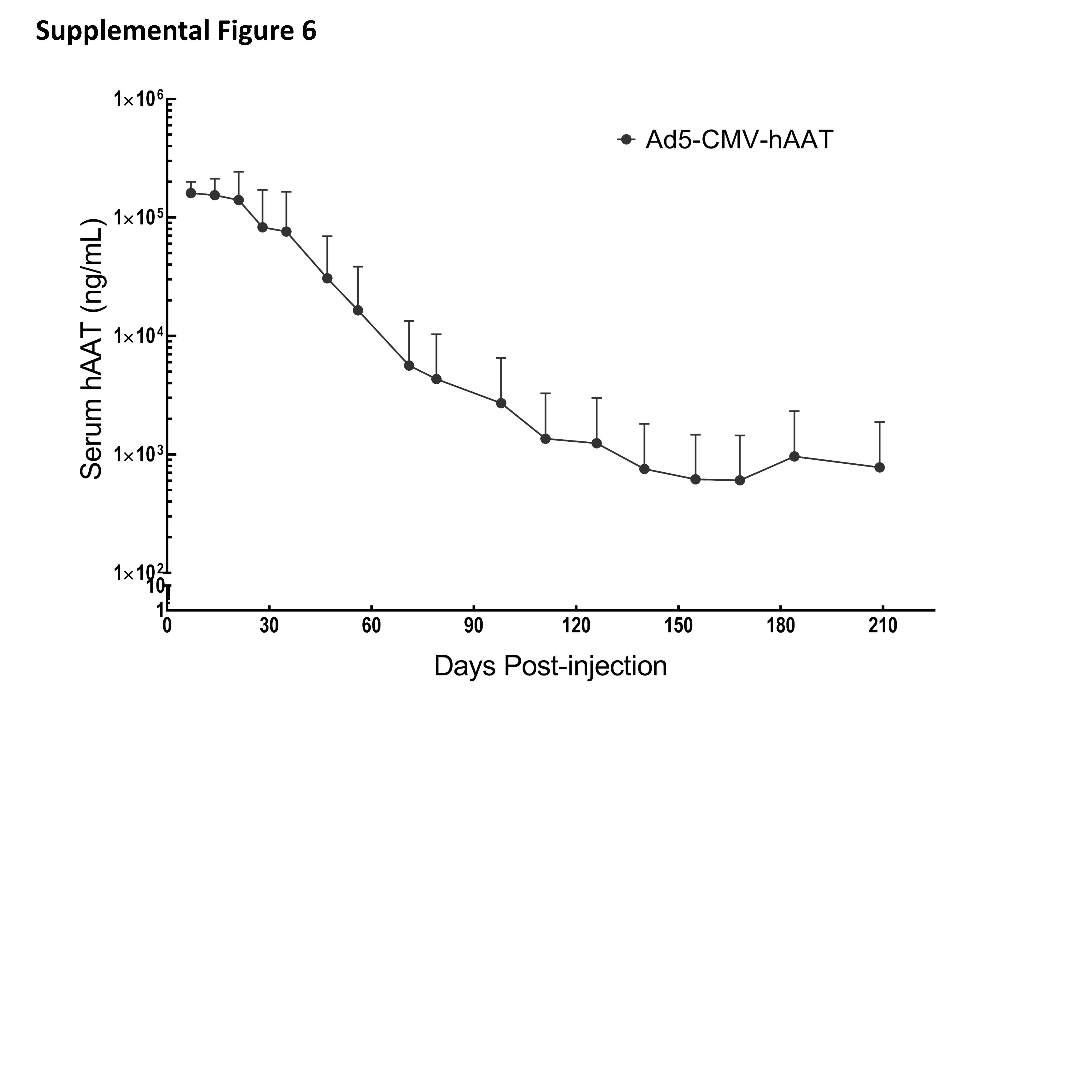

Supplement: Supplementary file 11 — Supplemental Figure 6(TIF 2490 kb) [file 41434_2018_3_MOESM11_ESM.tif]

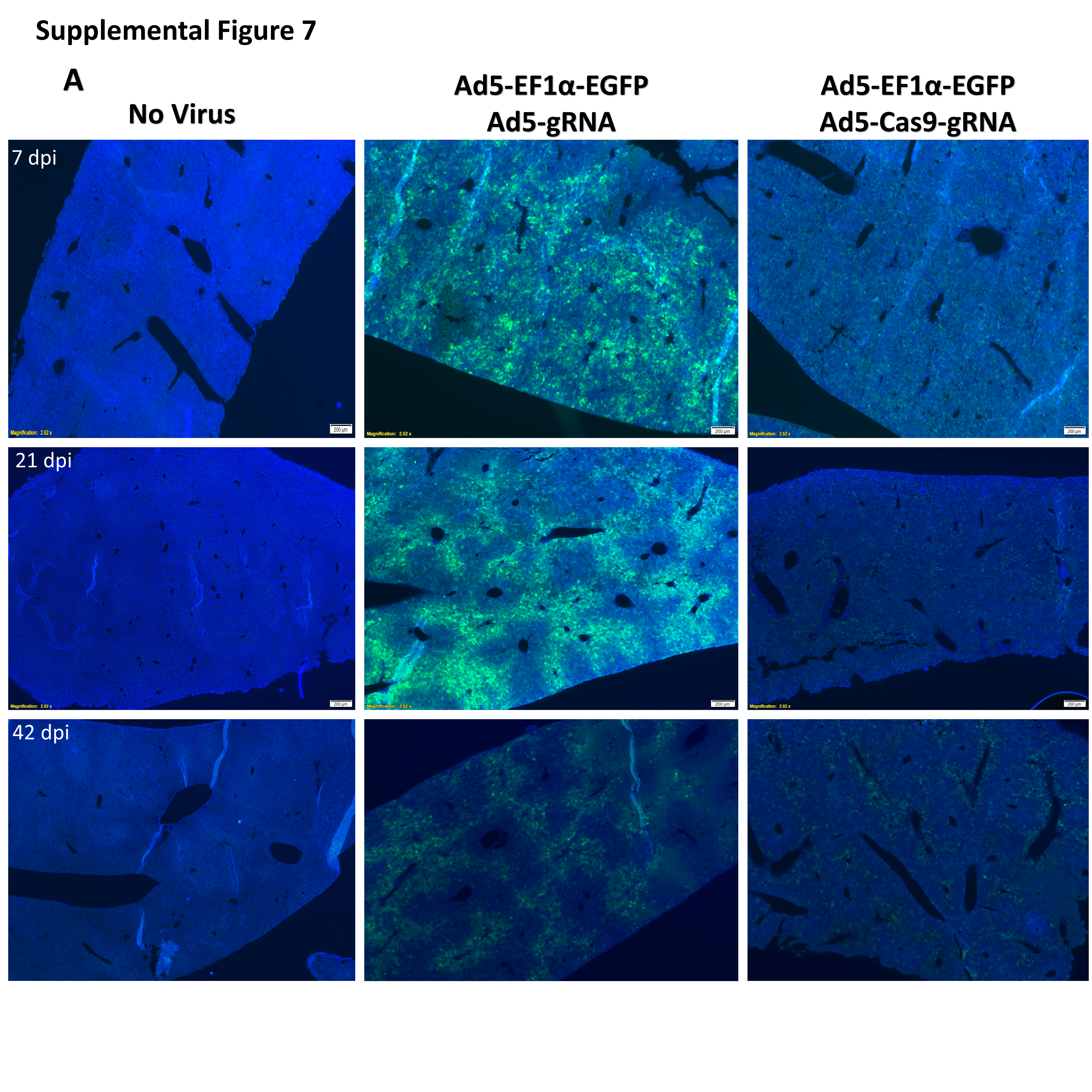

Supplement: Supplementary file 12 — Supplemental Figure 7(TIF 37101 kb) [file 41434_2018_3_MOESM12_ESM.tif]

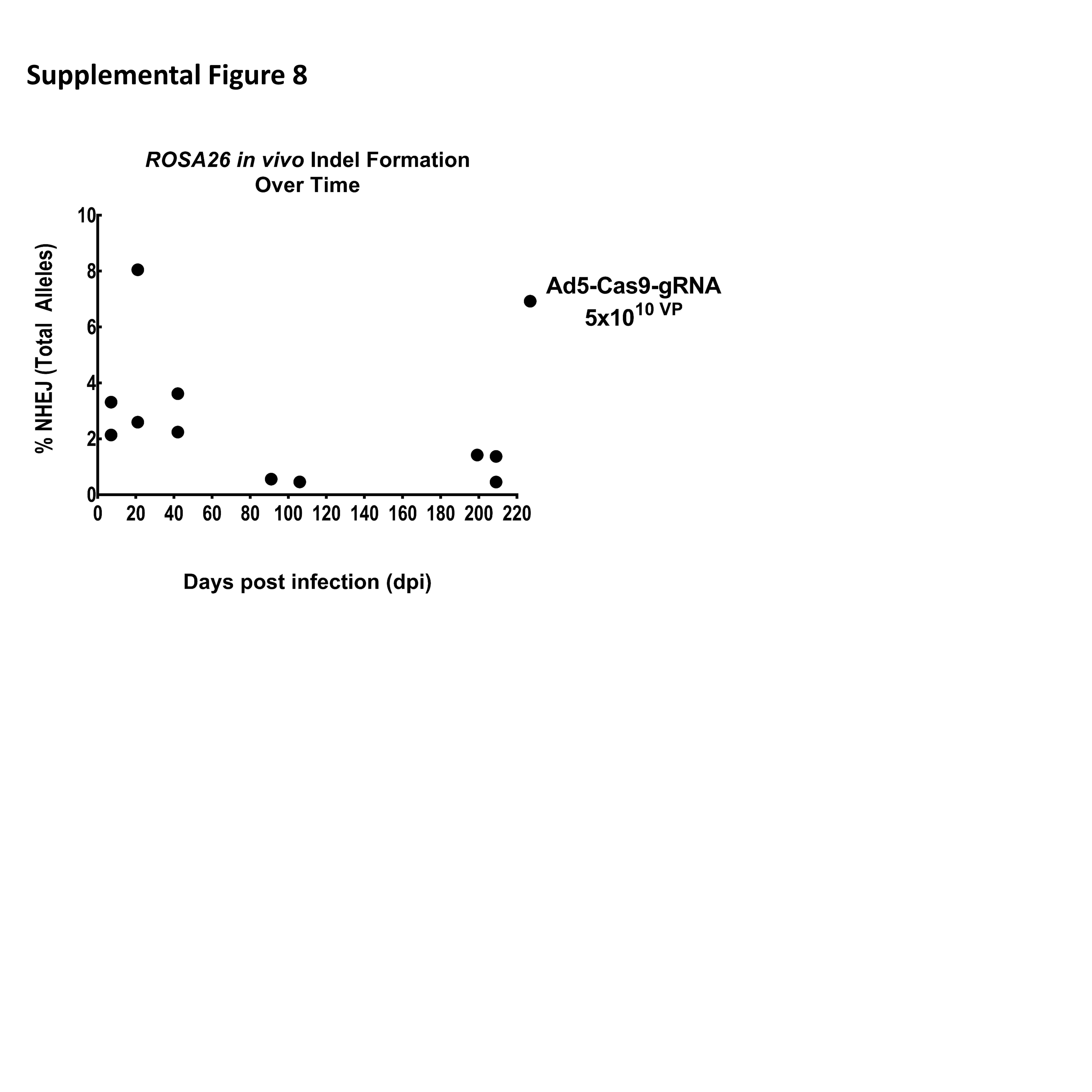

Supplement: Supplementary file 13 — Supplemental Figure 8(TIF 2287 kb) [file 41434_2018_3_MOESM13_ESM.tif]

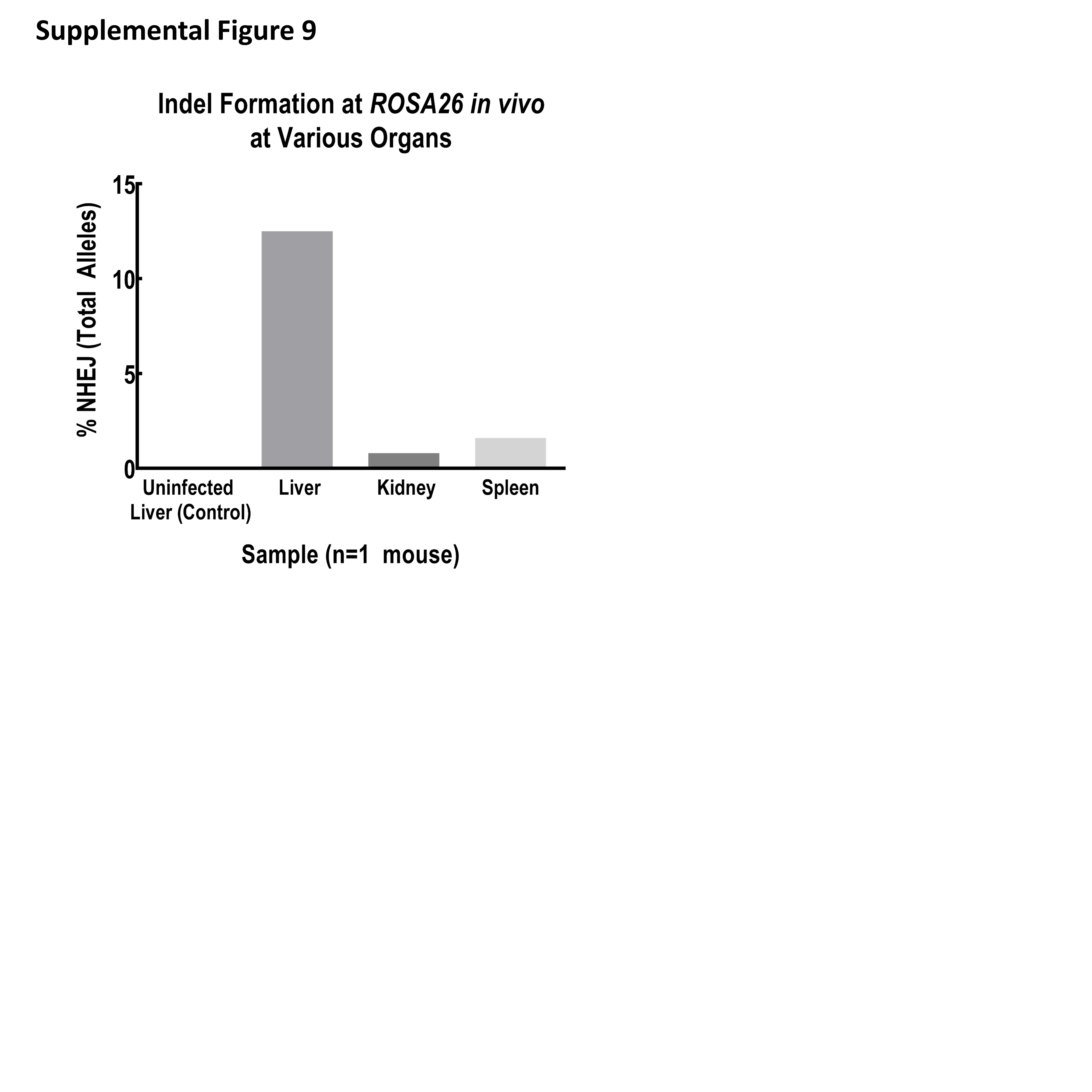

Supplement: Supplementary file 14 — Supplemental Figure 9(TIF 2337 kb) [file 41434_2018_3_MOESM14_ESM.tif]

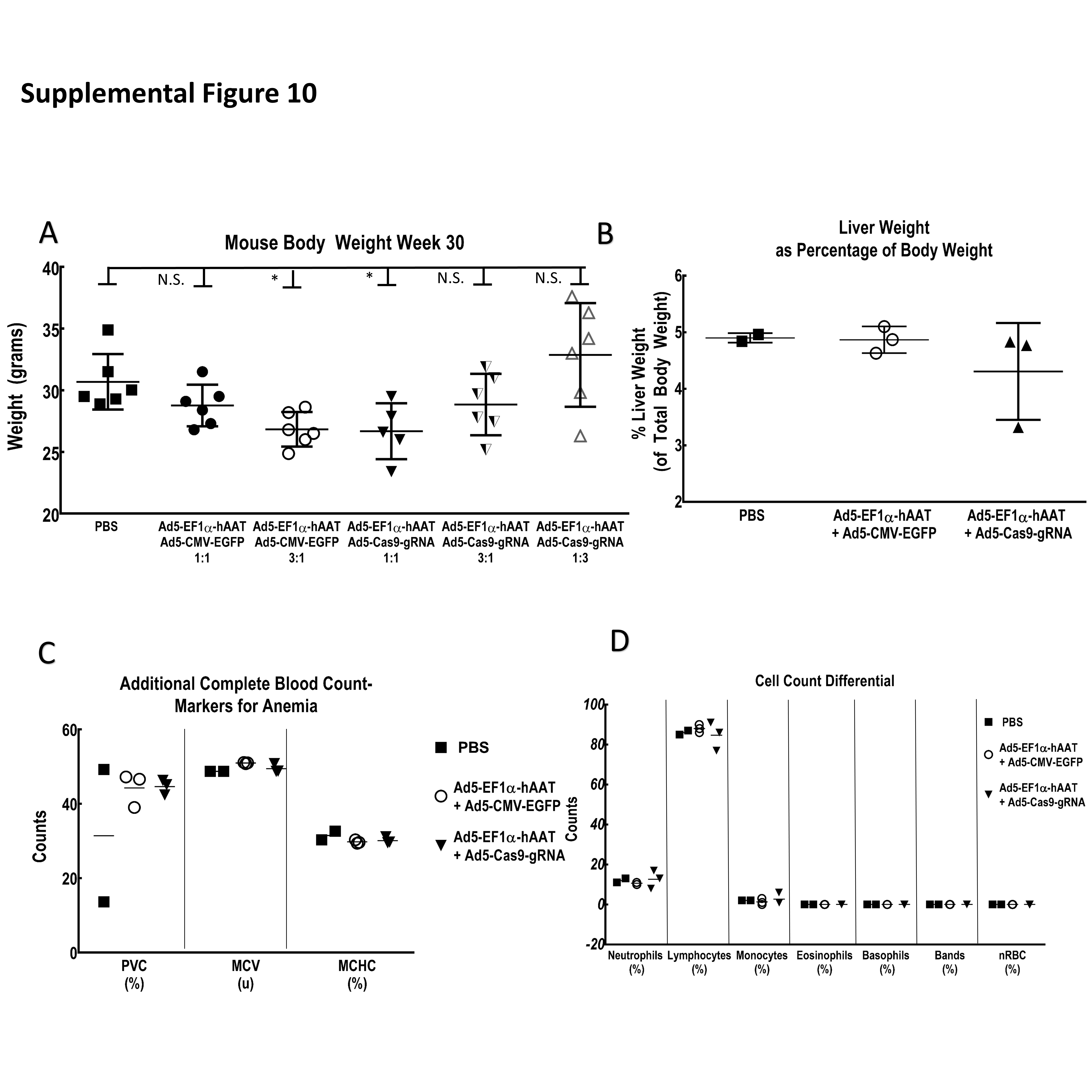

Supplement: Supplementary file 15 — Supplemental Figure 10(TIF 3880 kb) [file 41434_2018_3_MOESM15_ESM.tif]
